# Global trends in the burden of ischemic heart disease attributable to smoking from 1990 to 2021: A systematic analysis of the Global Burden of Disease Study 2021

**DOI:** 10.18332/tid/199931

**Published:** 2025-01-29

**Authors:** Yifei Wang, Qing Li, Lei Bi, Bin Wang, Tingting Lv, Ping Zhang

**Affiliations:** 1 Department of Cardiology, Beijing Tsinghua Changgung Hospital, School of Clinical Medicine, Tsinghua University, Beijing, China; 2 School of Clinical Medicine, Tsinghua University, Beijing, China

**Keywords:** ischemic heart disease, smoking, global burden of disease, socioeconomic characteristics, spatiotemporal trends

## Abstract

**INTRODUCTION:**

Ischemic heart disease (IHD) remains a leading cause of death and disability worldwide. This study evaluates the trends in IHD burden attributable to smoking, utilizing Global Burden of Disease Study 2021 (GBD 2021) data, across 204 countries and territories from 1990 to 2021. By examining age-standardized death rates (ASDR) and disability-adjusted life years (ASRDALYs), the study provides insights into the spatiotemporal variations associated with smokingattributable IHD in different sociodemographic regions.

**METHODS:**

Data on smoking-attributable IHD mortality and DALYs were obtained from the GBD 2021 database. This secondary analysis examined ASDR and ASRDALYs for IHD as primary outcomes, with active smoking as the primary exposure. Temporal trends were analyzed using estimated annual percentage changes (EAPCs). The burden was stratified by age, sex, and sociodemographic index (SDI) to identify disparities across regions.

**RESULTS:**

Over the last three decades, global ASDR and ASRDALYs for smokingrelated IHD have generally declined. High-SDI regions had the largest reductions, with EAPCs of -4.31 (95% CI: -5.73 – -2.87) and -4.02 (95% CI: -5.40 – -2.62), respectively. In contrast, low-SDI regions experienced slower declines, with EAPCs of -0.54 (95% CI: -1.41–0.33) and -0.80 (95% CI: -1.52 – -0.08), respectively. Older age groups and males consistently had a higher burden across all regions. Global death rates for populations aged 15–49, 50–74, and ≥75 years were 4.31, 46.57, and 142.52 per 100000, respectively. The global ASDR for males (30.24) was 8.54 times higher than that for females (3.54). Regional disparities were most pronounced in low- and middle-income areas, particularly in Eastern Europe and Central Asia, where smoking related IHD burden remains high.

**CONCLUSIONS:**

While global reductions in smoking-related IHD burden are encouraging, sustained disparities remain, particularly in low-SDI regions. Males and older populations continue to have a disproportionately higher burden, emphasizing the need for targeted interventions and sustained efforts to address these inequities.

## INTRODUCTION

Ischemic heart disease (IHD) is a leading cause of death and disability globally, representing a major public health challenge across various sociodemographic and economic groups^1^. According to data from the Global Burden of Disease Study 2021 (GBD 2021), IHD resulted in 8.99 million deaths and 188.3 million disability-adjusted life years (DALYs) in 2021, making it the leading cause of health loss among non-communicable diseases^2^. Effective screening and control of IHD and its risk factors are urgently needed to improve global health outcomes. Several risk factors contribute to IHD pathogenesis, including behavioral, metabolic, and environmental factors^1,3-5^. Among these risk factors, smoking remains one of the most significant modifiable behaviors, playing a crucial role in both the onset and progression of IHD^3,6-8^. Smoking exacerbates atherosclerosis and promotes thrombosis, thereby increasing the risk of myocardial infarction and other complications related to IHD, and significantly raises all-cause mortality (hazard ratio=1.92)^7,9^.

While global smoking prevalence has declined over the past few decades, largely due to public health initiatives, substantial differences in smoking rates still exist between regions, resulting in varied IHD burdens attributable to smoking^7^. High-SDI countries have implemented successful anti-smoking policies, leading to significant reductions in smoking rates and associated cardiovascular disease. In contrast, many low- and middle-SDI regions continue to face challenges with high smoking prevalence, especially among males, coupled with insufficient healthcare resources to support effective tobacco control efforts^10,11^.

This study aims to provide a comprehensive analysis of the global and regional trends in smoking-related IHD from 1990 to 2021, using data from the GBD 2021 dataset. By examining age-standardized death rates (ASDR) and disability-adjusted life years (ASRDALYs), and calculating estimated annual percentage changes (EAPCs), this study seeks to understand how smoking impacts IHD burden across different sociodemographic index (SDI) levels, age groups, and gender. The goal is to identify the disparities in smoking-attributable IHD and highlight areas and populations that require targeted public health interventions. Understanding these patterns is crucial for informing policies aimed at reducing the burden of smoking-related IHD and improving cardiovascular health outcomes globally.

## METHODS

### Study design and data source

This study was a secondary dataset analysis using the Global Burden of Disease Study 2021 (GBD 2021), which provides detailed epidemiological data on IHD and associated risk factors, including smoking, for 204 countries and territories from 1990 to 2021. Data were sourced from the Global Health Data Exchange (GHDx) platform (https://vizhub.healthdata.org/gbd-results/), managed by the Institute for Health Metrics and Evaluation (IHME). The GBD methodology follows the Guidelines for Accurate and Transparent Health Estimates Reporting (GATHER), ensuring that data collection, modeling, and estimates are reproducible and transparent^12^.

### Disease definition and measurement

IHD was defined using the International Classification of Diseases, 10th Revision codes I20–I25, which include angina, myocardial infarction, and chronic IHD. The burden of IHD attributable to smoking was measured using ASDR and ASRDALYs^13^. DALYs were calculated by summing years of life lost due to premature death and years lived with disability^14^. The primary outcomes of interest were smoking-attributable ASDR and ASRDALYs, with active smoking identified as the primary exposure.

The SDI was used to quantify a country’s level of development, incorporating data on fertility, education, and income per capita, with values ranging from 0 to 1. Countries and regions were categorized into five SDI quintiles (low, low-middle, middle, high-middle, and high) to explore the association between IHD burden and socioeconomic development. This classification enabled stratified analyses of IHD burden by SDI level.

### Attribution of smoking as a risk factor

The burden of smoking-related IHD was estimated using the GBD Comparative Risk Assessment (CRA) framework, which applies population attributable fractions (PAFs). PAFs were calculated based on the hypothetical reduction in disease burden if exposure to smoking were minimized to a theoretical minimum risk exposure level (TMREL). Relative risk estimates and smoking prevalence data were used to determine PAFs across age, sex, and regional groups. Notably, the analysis excluded secondhand smoke and focused solely on the burden attributable to active smoking.

### Statistical analysis

*Trend analysis using estimated annual percentage change (EAPC)*

To assess changes over time in smoking-related ASDR and ASRDALYs for IHD, estimated annual percentage changes (EAPCs) were calculated using a log-linear regression model: APC=100×(e^β^-1). The EAPC was derived from the slope (β) of the regression of the natural logarithm of ASDR or ASRDALYs on calendar year. A 95% confidence interval (CI) excluding zero indicated a statistically significant trend. EAPCs were calculated separately for each SDI level and gender to capture demographic-specific trends. During the analysis, annual time points from 1990 to 2021, totally 32 data points, were included.

*Joinpoint regression analysis*

Joinpoint regression was applied to identify significant changes in temporal trends. This method fits multiple linear segments to the data and detects points where the trend direction changes (joinpoints). Analyses were performed using Joinpoint software (version 5.3.0.0, National Cancer Institute, USA) for global, SDI regions, and sex groups. Annual percentage change (APC) was calculated for each segment, along with 95% CI. Trends were considered statistically significant if p<0.05, while p>0.05 indicated no significant change, suggesting a flat trend.

*Stratified analysis by age, sex, and SDI*

Analyses were stratified by age group, sex, and SDI quintile to examine disparities in smoking-attributable IHD burden. Age-specific trends were analyzed across 5-year intervals to detect variations in IHD burden among different age groups. Sex-specific and SDI-specific analyses were further stratified by geographical region, providing insights into how socioeconomic and demographic factors influence IHD trends associated with smoking.

*Uncertainty and sensitivity analysis*

To account for data variability, 95% uncertainty intervals (UIs) were calculated for ASDR and ASRDALYs^15^. These UIs were derived using 1000 Monte Carlo simulations, which incorporate multiple sources of uncertainty, including variations in input data, model parameters, and adjustments for covariates. The 95% UI represents the range within which the true value is expected to fall with 95% probability, providing a comprehensive measure of uncertainty around each estimate. Sensitivity analyses were conducted by adjusting for potential confounders in smoking prevalence and regional variations in healthcare access and policy.

### Software and tools

All statistical analyses and data visualization were conducted using Joinpoint (version 5.3.0.0), R (version 4.1.2) and GraphPad prism 9.

## RESULTS

### Global burden of IHD and attributable risk factors

The analysis of the global IHD burden in 2021, using ASDR and ASRDALYs, reveals significant geographical variability. The global ASDR for IHD was 108.97 (95% CI: 99.60–115.38) per 100000 people, while ASRDALYs was 2212.16 (95% CI: 2075.54–2327.61) per 100000 people (Supplementary file Table 1), and Central Asia, Eastern Europe, and East Asia had the highest IHD burden (Supplementary file Figure 1). Within these regions, countries like Mongolia, Kazakhstan, and Ukraine stood out as hotspots. In contrast, high-income regions such as North America, Western Europe, and Australasia reported significantly lower rates.

From 1990 to 2021, the global IHD burden, measured by ASDR and ASRDALYs, showed a gradual decline, particularly in high-income regions. However, certain areas, including East Asia, South Asia, and Southern Sub-Saharan Africa, continued to experience rising mortality rates (Supplementary file Table 1). And smoking remains a major behavioral risk factor for IHD, particularly affecting males (Supplementary file Figures 2 and 3). Men have historically had higher smoking rates, which have led to a disproportionate burden of smoking-related IHD.

### Global and regional burden of IHD attributable to smoking

Smoking is a well-established behavioral risk factor for IHD and significantly contributes to the burden in certain regions, with notable differences across countries. According to the GBD 2021 database, the global burden of smoking-related IHD in 2021 was 15.76 (95% CI: 13.33–18.39) per 100000 people for ASDR and 408.78 (95% CI: 351.16–469.81) per 100000 people for ASRDALYs (Table 1). Eastern Europe, Central Asia, and parts of Southeast Asia had the highest burden. The Republic of Nauru had the highest ASDR for smoking at 79.76 (95% CI: 59.34–104.41) per 100000 people and the highest ASRDALYs at 2653.48 (95% CI: 1968.44–3517.89) per 100000 people.

**Table 1:** Global distribution and EAPC of IHD burden attributable to smoking (both sexes)

*Location*	*ASDR (95% UI)*		*ASRDALYs (95% UI)*		*EAPC (95% CI)*	
	*1990*	*2021*	*1990*	*2021*	*ASDR*	*ASRDALYs*
**Global**	26.93 (22.96–31.00)	15.76 (13.33–18.39)	691.94 (606.61–781.27)	408.78 (351.16–469.81)	-1.81 (-2.31 – -1.30)	-1.81 (-2.45 – -1.16)
**SDI**						
Low SDI	13.40 (10.90–16.12)	11.28 (9.07–13.74)	367.16 (300.88–442.73)	293.91 (241.48–351.66)	-0.54 (-1.41–0.33)	-0.80 (-1.52 – -0.08)
Low-middle SDI	23.44 (19.46–27.55)	20.35 (17.00–23.81)	648.65 (552.37–758.00)	543.48 (456.47–627.88)	-0.32 (-0.99–0.35)	-0.45 (-1.04–0.15)
Middle SDI	21.73 (18.21–25.24)	16.88 (13.90–20.05)	565.13 (489.40–645.76)	417.30 (350.81–483.51)	-0.74 (-1.41 – -0.06)	-0.94 (-1.45 – -0.43)
High-middle SDI	30.06 (26.00–34.15)	19.40 (16.06–22.95)	805.27 (711.08–898.51)	486.96 (416.30–560.00)	-1.67 (-4.55–1.30)	-1.98 (-5.05–1.19)
High SDI	31.89 (27.23–36.92)	9.00 (7.49–10.58)	786.79 (688.47–891.64)	238.93 (203.93–275.41)	-4.31 (-5.73 – -2.87)	-4.02 (-5.40 – -2.62)
**Region**						
Andean Latin America	8.50 (6.81–10.08)	4.53 (3.40–5.86)	216.68 (178.41–255.70)	119.42 (91.68–151.94)	-2.24 (-5.95–1.61)	-2.11 (-5.66–1.56)
Australasia	26.62 (22.33–31.14)	4.49 (3.69–5.46)	668.23 (571.67–771.47)	119.30 (101.52–140.62)	-5.87 (-7.14 – -4.57)	-5.65 (-7.14 – -4.13)
Caribbean	27.64 (23.06–32.73)	13.80 (10.93–17.05)	706.42 (610.19–817.49)	366.65 (297.13–446.33)	-2.35 (-3.80 – -0.88)	-2.21 (-3.80 – -0.60)
Central Asia	40.20 (34.71–46.02)	30.35 (25.25–35.50)	1137.62 (997.89-1276.41)	766.98 (640.34–888.80)	-1.24 (-4.83–2.49)	-1.76 (-5.68–2.32)
Central Europe	50.64 (43.62–57.30)	18.44 (15.34–21.70)	1388.08 (1222.63–1541.26)	486.36 (412.26–561.17)	-3.60 (-4.64 – -2.55)	-3.74 (-4.79 – -2.67)
Central Latin America	16.28 (13.64–18.96)	8.36 (6.67–10.09)	409.34 (351.35–468.41)	219.36 (179.98–263.13)	-2.49 (-4.10 – -0.86)	-2.37 (-4.18 – -0.53)
Central Sub-Saharan Africa	9.25 (6.78–12.22)	7.14 (5.28–9.47)	260.76 (191.39–346.49)	203.95 (147.63–269.13)	-0.92 (-3.44–1.66)	-0.87 (-3.22–1.53)
East Asia	18.17 (14.92–21.89)	19.01 (14.62–24.18)	435.68 (360.02–524.21)	417.54 (324.75–522.58)	0.59 (-1.73–2.98)	0.24 (-1.59–2.09)
Eastern Europe	42.72 (37.39–47.95)	34.79 (28.96–40.79)	1225.95 (1093.00–1363.13)	970.33 (820.32–1128.79)	-1.23 (-7.60–5.57)	-1.45 (-8.00–5.57)
Eastern Sub-Saharan Africa	6.47 (5.21–7.90)	5.61 (4.36–7.03)	175.26 (142.98–213.64)	152.69 (120.04–189.38)	-0.76 (-1.73–0.22)	-0.73 (-1.65–0.21)
High-income Asia Pacific	12.25 (10.32–14.35)	3.88 (3.24–4.54)	289.95 (252.04–325.92)	104.95 (89.69–119.91)	-3.78 (-4.43 – -3.13)	-3.37 (-4.07 – -2.66)
High-income North America	38.03 (32.18–44.42)	11.90 (9.73–14.05)	955.27 (829.74–1089.42)	310.64 (261.21–360.86)	-4.10 (-5.81 – -2.36)	-3.92 (-5.56 – -2.25)
North Africa and Middle East	44.52 (37.64–51.97)	27.55 (22.11–33.22)	1191.45 (1025.58–1370.39)	716.37 (582.15–857.96)	-1.70 (-2.13 – -1.26)	-1.77 (-2.14 – -1.41)
Oceania	29.87 (23.49–37.23)	25.19 (19.62–31.83)	940.01 (739.96–1182.73)	803.84 (634.22–1014.89)	-0.57 (-0.92 – -0.23)	-0.53 (-0.89 – -0.16)
South Asia	24.03 (19.56–28.89)	19.61 (16.07–23.52)	671.69 (560.14–795.87)	514.11 (424.34–606.68)	-0.57 (-1.46–0.32)	-0.81 (-1.52 – -0.09)
Southeast Asia	21.78 (18.13–25.60)	18.37 (15.32–21.61)	584.00 (491.26–673.02)	504.41 (423.81–596.67)	-0.60 (-1.26–0.07)	-0.49 (-0.91 – -0.07)
Southern Latin America	21.30 (18.22–24.72)	7.02 (5.97–8.26)	604.58 (526.52–688.15)	204.01 (176.56–234.38)	-3.34 (-4.02 – -2.65)	-3.32 (-3.97 – -2.66)
Southern Sub-Saharan Africa	13.66 (11.02–16.42)	8.26 (6.79–9.86)	384.28 (323.14–448.08)	235.99 (197.70–278.16)	-1.80 (-4.62–1.11)	-1.72 (-4.39–1.02)
Tropical Latin America	31.39 (26.54–36.88)	9.93 (8.15–12.12)	844.61 (733.63–959.43)	275.72 (229.88–331.33)	-3.79 (-5.26 – -2.30)	-3.76 (-4.96 – -2.54)
Western Europe	30.54 (26.13–35.38)	6.69 (5.57–7.95)	742.19 (647.44–840.25)	169.45 (144.92–195.36)	-5.11 (-6.28 – -3.93)	-4.93 (-6.01 – -3.83)
Western Sub-Saharan Africa	5.03 (3.85–6.28)	4.22 (3.22–5.25)	132.82 (101.71–165.88)	112.21 (86.09–138.99)	-0.67 (-2.57–1.27)	-0.66 (-2.77–1.49)

The ASDR (age-standardized death rates) and ASRDALYs (age-standardized disability-adjusted life years) per 100000 population of IHD (ischemic heart disease) attributable to smoking among both sexes across global regions, SDI (sociodemographic index) levels, and specific geographical regions for 1990 and 2021 are summarized. Temporal trends from 1990 to 2021 are represented by the estimated annual percentage change (EAPC) of ASDR and ASRDALYs, with corresponding 95% confidence intervals (CI). Positive EAPC values indicate an increasing trend, while negative EAPC values denote a decreasing trend. UI: uncertainty interval.

At the regional level, Central Asia, Eastern Europe, North Africa and the Middle East, and Oceania had the greatest burden of smoking-related IHD in 2021. Central Asia had the highest ASDR at 30.35 (95% CI: 25.25–35.50) per 100000 people, while Oceania had the highest ASRDALYs at 803.84 (95% CI: 634.22–1014.89) per 100000 people. In contrast, high-SDI regions showed lower mortality rates from smoking-related IHD. The high-income Asia-Pacific region had the lowest ASDR at 3.88 (95% CI: 3.24–4.54) per 100000 people and the lowest ASRDALYs at 104.95 (95% CI: 89.69–119.91) per 100000 people.

Gender also plays a significant role in the burden of smoking-related IHD, with males experiencing a disproportionately higher impact. This trend is consistent across most regions, particularly in Eastern Europe and Central Asia, where smoking prevalence is significantly higher among males.

### Temporal trends of IHD burden attributable to smoking from 1990 to 2021

Globally, the burden of IHD has shown a consistent decline over the past three decades (Supplementary file Figure 4). However, this overall trend conceals significant disparities across regions and countries. High-SDI regions have experienced the most substantial reductions in IHD mortality and morbidity. However, low- and middle-SDI regions continue to face a relatively high burden, where declines in ASDR and ASRDALYs have been less pronounced or have plateaued.

The trend in smoking-related IHD burden follows a similar pattern. The EAPC data indicate a general decrease in smoking-related IHD burden from 1990 to 2021, although regional differences remain (Supplementary file Figure 5). High-SDI regions have seen the most pronounced decreases, with EAPCs of -4.31 (95% CI: -5.73 – -2.87) for ASDR and -4.02 (95% CI: -5.40 – -2.62) for ASRDALYs (Table 1). In contrast, low- and middle-SDI regions have shown more modest reductions. Australasia had the highest reductions over the past three decades, with EAPCs of -5.87 (95% CI: -7.14 – -4.57) for ASDR and -5.65 (95% CI: -7.14 – -4.13) for ASRDALYs.

**Figure 1:**
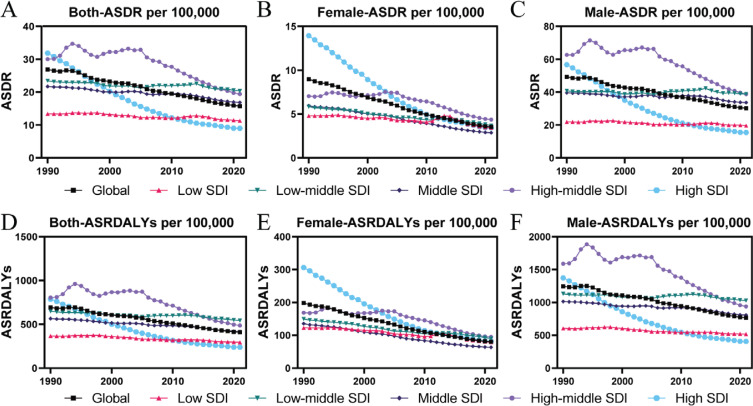
Temporal trends in smoking-attributable IHD burden. The temporal trends in the smoking-attributable IHD burden from 1990 to 2021, represented by ASDR per 100000 population for both sexes (A), females (B), and males (C), and by ASRDALYs per 100000 population for both sexes (D), females (E), and males (F), stratified by sociodemographic index (SDI) levels

In high- and high-middle-SDI countries, both sexes experienced significant decreases in smoking-related IHD ASDR and ASRDALYs over the past 30 years (Figure 1). In 1990, high-SDI countries had an ASDR of 31.89 (95% CI: 27.23–36.92) and ASRDALYs of 786.79 (95% CI: 688.47– 891.64), which decreased significantly to 9.00 (95% CI: 7.49–10.58) for ASDR and 238.93 (95% UI: 203.93–275.41) by 2021. The trend was similar for both males and females.

In low-, low-middle-, and middle-SDI regions, trends were more varied. While some reduction in smoking-related IHD burden was observed, the rate of decline was much slower compared to high-SDI regions. Among males, ASDR and ASRDALYs decreased only slightly, whereas females showed a more noticeable decline. In these regions, the EAPC for ASDR among females ranged from -0.85 (95% CI: -2.85–1.19) in low-SDI regions to -2.47 (95% CI: -3.53 – -1.41) in middle-SDI regions. For ASRDALYs, the EAPC ranged from -1.36 (95% CI: -2.67 – -0.04) to -2.58 (95% CI: -3.18 – -1.98) (Supplementary file Table 2). Among males, the EAPC for ASDR ranged from -0.01 (95% CI: -0.73–0.72) to -0.40 (95% CI: -1.17–0.37), while the EAPC for ASRDALYs ranged from -0.18 (95% CI: -0.83–0.48) to -0.67 (95% CI: -1.26 – -0.07) (Supplementary file Table 3).

Joinpoint analysis of ASDR (Table 2) and ASRDALYs (Supplementary file Table 4) revealed non-linear declines in smoking-related IHD burden. Globally, a significant reduction began in 1994 (p<0.001). While middle-SDI regions followed a similar timeline, high-SDI and high-middle SDI regions experienced significant declines as early as 1990. However, in low-SDI and low-middle SDI regions, no significant decline was observed even by 2021 (p>0.05). Further analysis indicated that in low-SDI and low-middle SDI regions, smoking-related IHD burden continued to decline significantly among females. In contrast, the burden among males remained largely stagnant, with flat trends observed across various time segments (p>0.05) (Table 2).

**Table 2:** Trends in ASDR of IHD attributable to smoking by sex and SDI regions from 1990 to 2021

*Location*	*All*			*Females*			*Males*		
	*Time point*	*APC (95% CI)*	*p*	*Time point*	*APC (95% CI)*	*p*	*Time point*	*APC (95% CI)*	*p*
Global	1990–1994 1994–1998* 1998–2003* 2003–2021*	-0.21 (-0.69–0.37) -2.67 (-3.32 – -2.17) -1.16 (-1.50 – -0.44) -1.99 (-2.07 – -1.94)	0.396 <0.001 <0.001 <0.001	1990–1994* 1994–2004* 2004–2007* 2007–2021*	-1.98 (-2.40 – -1.18) -2.85 (-3.02 – -2.71) -4.14 (-4.40 – -3.43) -3.11 (-3.19 – -2.95)	<0.001 <0.001 <0.001 <0.001	1990–1994 1994–1998* 1998–2003 2003–2021*	-0.11 (-0.62–0.62) -2.69 (-3.48 – -2.13) -0.87 (-1.26–0.04) -1.82 (-1.91 – -1.75)	0.710 <0.001 0.057 <0.001
Low SDI	1990–1997 1997–2005 2005–2010 2010–2014 2014–2018 2018–2021	0.43 (-0.03–0.81) -1.25 (-1.82–0.78) -0.57 (-1.36–0.33) 1.63 (-0.53–2.39) -2.46 (-3.15–1.47) -0.99 (-1.93–0.08)	0.054 0.104 0.106 0.094 0.096 0.070	1990–1995 1995–2010* 2010–2013* 2013–2021*	0.29 (-0.63–2.53) -1.11 (-1.60 – -0.94) 5.30 (2.74–6.34) -4.50 (-5.02 – -4.05)	0.516 0.014 0.014 <0.001	1990–1998 1998–2005 2005–2011 2011–2014 2014–2021*	0.40 (-0.45–0.86) -1.32 (-2.23–1.14) -0.16 (-1.48–0.80) 1.47 (-1.98–1.99) -1.17 (-1.76 – -0.16)	0.095 0.136 0.347 0.131 0.042
Low-middle SDI	1990–2005* 2005–2014 2014–2017 2017–2021	-0.57 (-0.81 – -0.29) 0.45 (-0.93–0.93) -2.15 (-2.68–0.85) -0.65 (-1.42–0.57)	0.036 0.208 0.255 0.205	1990–1998* 1998–2001* 2001–2011* 2011–2014 2014–2021*	-1.03 (-1.26 – -0.57) -2.55 (-2.94 – -1.57) -1.31 (-1.56 – -0.47) 1.37 (-0.17–1.84) -2.72 (-3.09 – -2.43)	0.018 0.006 0.044 0.060 <0.001	1990–2005* 2005–2014 2014–2017 2017–2021	-0.27 (-0.43 – -0.14) 0.83 (-0.21–1.19) -2.13 (-2.68–0.94) -0.24 (-1.08–1.09)	0.026 0.073 0.114 0.608
Middle SDI	1990 –1995 1995–2000* 2000–2004* 2004–2007* 2007–2011* 2011–2021*	-0.41 (-0.65–0.02) -1.27 (-1.84 – -1.02) 0.38 (0.03–0.93) -1.70 (-2.07 – -1.12) 0.53 (0.10–1.03) -1.59 (-1.70 – -1.48)	0.057 0.005 0.037 0.006 0.016 <0.001	1990–1996* 1996–1999* 1999–2004* 2004–2007* 2007–2011* 2011–2017* 2017–2021*	-1.11 (-1.23 – -0.93) -2.72 (-2.95 – -2.24) -1.33 (-1.49 – -1.01) -4.14 (-4.43 – -3.78) -2.27 (-2.53 – -1.84) -3.39 (-3.76 – -3.22) -2.16 (-2.49 – -1.60)	<0.001 <0.001 <0.001 <0.001 <0.001 <0.001 <0.001	1990–2001* 2001–2004* 2004–2007* 2007–2011* 2011–2021*	-0.68 (-0.86 – -0.56) 1.09 (0.27–1.49) -1.38 (-1.88 – -0.65) 1.08 (0.51–1.87) -1.36 (-1.54 – -1.19)	0.002 0.020 0.012 0.013 <0.001
High-middle SDI	1990–1994* 1994–1998* 1998–2004* 2004 –2021*	4.08 (3.02–5.26) -2.62 (-4.02 – -1.46) 1.15 (0.39–2.47) -3.23 (-3.39 – -3.08)	<0.001 <0.001 0.002 <0.001	1990–1994* 1994–1998* 1998–2004* 2004–2011* 2011–2021*	1.82 (1.06–3.04) -1.55 (-2.67 – -0.70) 1.13 (0.62–2.21) -2.69 (-3.12 – -1.66) -3.68 (-4.36 – -3.42)	<0.001 0.001 0.001 0.006 <0.001	1990–1994* 1994–1998* 1998–2004* 2004–2021*	3.77 (2.80–4.81) -2.87 (-4.18 – -1.77) 0.97 (0.26–2.06) -3.28 (-3.43 – -3.15)	<0.001 <0.001 0.004 <0.001
High SDI	1990–1994* 1994–2010* 2010–2014* 2014–2021*	-3.67 (-4.05 – -3.11) -4.97 (-5.05 – -4.91) -3.43 (-4.58 – -2.84) -2.44 (-2.64 – -2.04)	<0.001 <0.001 <0.001 <0.001	1990–1993* 1993–1999* 1999–2011* 2011–2021*	-3.44 (-4.24 – -2.44) -4.51 (-5.96 – -4.27) -5.75 (-6.00 – -3.24) -3.24 (-3.43 – -2.95)	<0.001 <0.001 <0.001 <0.001	1990–1994* 1994–2009* 2009–2014* 2014–2021*	-3.98 (-4.35 – -3.44) -5.12 (-5.20 – -5.06) -3.73 (-4.28 – -3.22) -2.43 (-2.63 – -2.13)	<0.001 <0.001 <0.001 <0.001

The joinpoint regression results for ASDR (per 100000 population) trends of IHD attributable to smoking across SDI regions and stratified by sex from 1990 to 2021 are shown. The time points represent the breakpoints where significant changes in the trends occurred, with APC (annual percent change) and 95% CI provided. Positive APC values indicate an increasing trend, while negative APC values denote a decreasing trend.

* Statistically significant APCs (p<0.05); statistically significantly increasing or decreasing trend; p>0.05 suggests no significant change (stable trend).

### Sex-specific IHD burden and trends attributable to smoking

**Figure 2:**
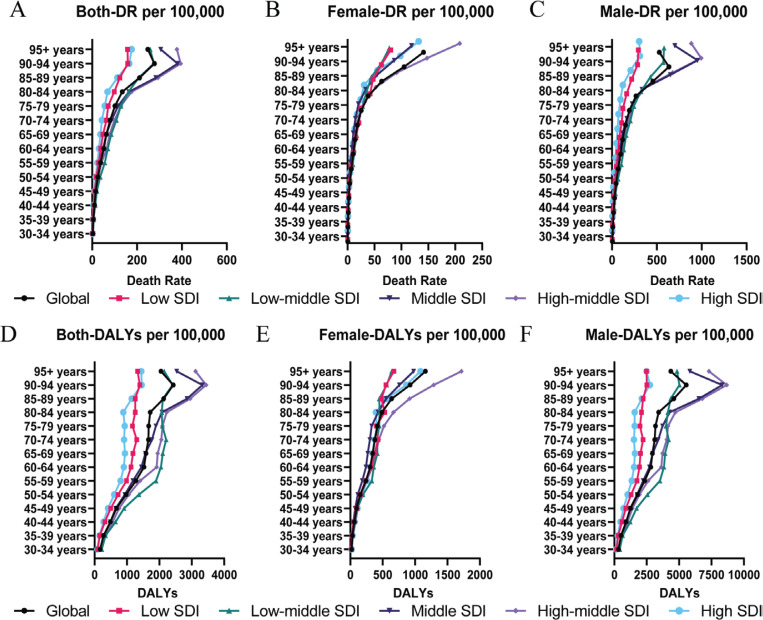
Age- and sex- specific IHD burden attributable to smoking in 2021 Global age- and sex-specific death rates (DR per 100000 population) and disability-adjusted life years (DALYs per 100000 population) for IHD attributable to smoking in 2021. Death rate trends are presented for both sexes (A), females (B), and males (C), while DALY trends are shown for both sexes (D), females (E), and males (F), across different age groups and SDI regions

The death rate (DR) and DALYs attributable to smoking show similar trends between males and females across all age categories (Figure 2). Globally in 2021, the ASDR and ASRDALYs for smoking-related IHD were significantly higher among males at 30.24 (95% CI: 25.38–35.58) and 768.01 (95% CI: 661.43–884.19) per 100000 population, compared to females at 3.54 (95% CI: 2.83–4.32) and 80.19 (95% CI: 66.48–95.81) (Supplementary file Tables 2 and 3). Across low, low-middle, middle, highmiddle, and high SDI regions, the male-to-female ratio of ASDR was 5.75, 10.81, 11.74, 8.87, and 4.53, respectively, while the ratio for ASRDALYs was 6.53, 11.38, 12.70, 9.90, and 4.99, respectively. At each SDI level, males consistently have a higher burden of smoking-attributable IHD compared to females, especially in middle-aged and older populations (Figure 2).

Among females, high-middle SDI regions showed the highest DR and DALYs in those aged ≥70 years, whereas low-middle SDI regions showed the highest rates in the 30–69 years age groups (Figures 2B and 2E). For males, high-middle SDI regions exhibited the highest burden in the age group of 75–95 years, while low-middle SDI regions had the highest rates in the age range of 30–74 years (Figures 2C and 2F). The upward trend in DR and DALYs with age is steeper in males, reflecting the cumulative impact of long-term smoking. Although females also experienced an age-related increase in IHD burden, the rise was less pronounced, indicating lower lifetime smoking exposure, particularly in high-SDI countries. Notably, DR and DALYs peaked for males in the age group of 90–95 years across all SDI regions, while they continued to increase in females beyond this age. This difference may be due to higher cumulative exposure among males, which could lead to earlier mortality before reaching the oldest age groups.

### Age-specific IHD burden and trends attributable to smoking

Age was another important risk factor for smoking related IHD burden. The age-specific burden of IHD showed significant variation from 1990 to 2021 across different SDI regions and age groups. The global DR and DALYs decreased considerably over this period, especially among older populations (Supplementary file Figures 6 and 7). However, this decline was primarily driven by high-SDI regions, while other regions showed only limited improvement.

**Figure 3:**
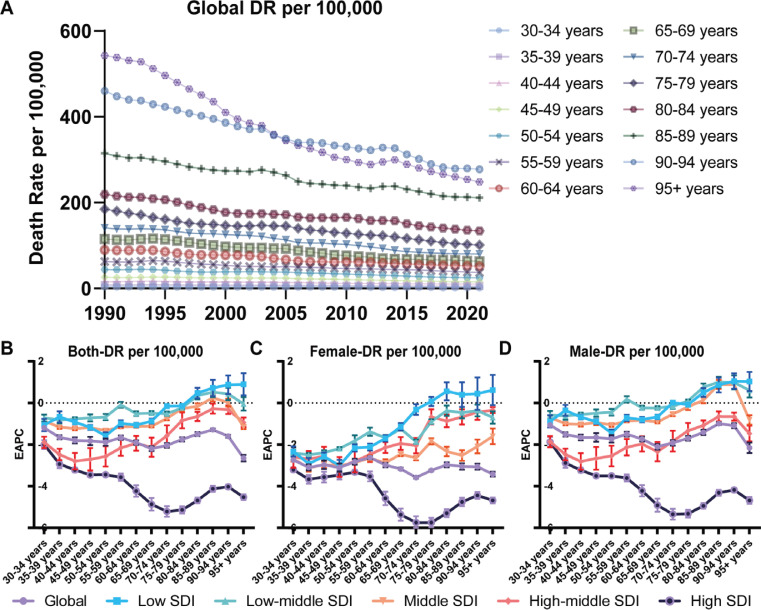
Temporal trends of global DR for IHD burden attributable to smoking across age groups (A) The global death rates (DR per 100000 population) of IHD attributable to smoking from 1990 to 2021 are shown, stratified by age groups (B–D). The EAPC of DR from 1990 to 2021 for both sexes (B), females (C), and males (D), across different age groups. Error bars indicate the 95% CI

**Figure 4:**
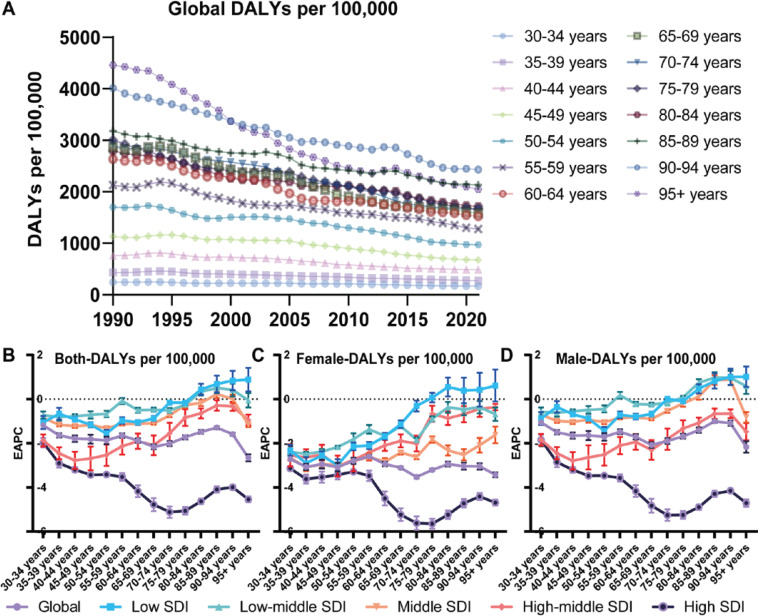
Temporal trends of global DALYs for IHD burden attributable to smoking across age groups (A) The global death rates (DALYs per 100000 population) of IHD attributable to smoking from 1990 to 2021 are shown, stratified by age groups (B–D). The EAPC of DALYs from 1990 to 2021 for both sexes (B), females (C), and males (D), across different age groups. Error bars indicate the 95% CI

Smoking-attributable DR and DALYs for IHD followed a consistent global trend. In 2021, DR for the populations aged 15–49, 50–74, and ≥75 years were 4.31 (95% CI: 3.73–4.92), 46.57 (95% CI: 39.63–53.52), and 142.52 (95% CI: 108.97–177.45) per 100000, respectively (Figures 3A and 4A), and a global decline in smoking-attributable DR and DALYs across all age groups was observed (Figures 3A and 4A). However, the IHD burden attributable to smoking varied significantly across SDI regions (Supplementary file Figures 8 and 9). High-SDI regions led these declines, with an EAPC of -4.36 (95% CI: -4.21 – -4.50) for DR and -4.57 (95% CI: -4.42 – -4.72) for DALYs in the age group of ≥75 years (Figures 3B and 4B, Supplementary file Figure 10). Middle-aged populations in high-SDI regions also experienced substantial declines, though at a slower rate compared to older groups.

In contrast, low-SDI regions demonstrated much slower progress. DR and DALYs for smoking-related IHD showed minimal reductions or even slight increases in certain age groups. For example, among populations aged ≥75 years in low-SDI regions, the burden slightly increased over the past three decades. The EAPC for DR among females in this group was 0.45 (95% CI: 0.06–0.83), while for males, it was 0.39 (95% CI: 0.24–0.54) (Figures 3C and 3D, Supplementary file Figure 10). Similarly, the EAPC for DALYs among females was 0.34 (95% CI: -0.004–0.68), and for males it was 0.22 (95% CI: 0.09–0.34) (Figures 4C and 4D, Supplementary file Figure 10).

These findings underscore the growing disparities in smoking-attributable IHD outcomes. While high-SDI regions have achieved significant progress in reducing the burden across all age groups, low-SDI populations – particularly older age groups –are not benefitting equally from global health advancements. Addressing these inequities is critical to ensuring more equitable health outcomes worldwide.

## DISCUSSION

Tobacco use is a known risk factor for increasing the risk of major adverse cardiovascular events related to IHD through multiple mechanism such as inflammation, endothelial dysfunction, smooth muscle cell proliferation, and atherosclerotic plaque disruption^16-19^. To reduce the health burden and economic costs of smoking, various policies – including taxation, announcements, and education – have been implemented, resulting in significant reductions in smoking prevalence: 27.5% among males and 37.7% among females^20,21^. However, the effectiveness of tobacco control policies varies across regions, leading to disparities in the trends of smoking-related IHD burden.

This study provides a detailed analysis of global trends in IHD burden attributable to smoking, using GBD 2021 data to examine changes by age, sex, and SDI from 1990 to 2021. Our findings show a global decline in ASDR and ASRDALYs for smoking-attributable IHD, with significant reductions in high-SDI regions. However, disparities persist across SDI levels and between gender groups, highlighting the need for targeted interventions to address ongoing inequalities.

Consistent with previous research, our study shows a decline in smoking-related IHD mortality and morbidity worldwide. High-SDI regions, which have implemented comprehensive anti-smoking measures and effective healthcare interventions, exhibit the greatest reductions in ASDR and ASRDALYs. The trend is consistent with the successful control of tobacco usage in high-SDI regions^22,23^. For instance, Western Europe and North America have successfully reduced IHD burden through sustained public health initiatives, leading to notable declines across all age groups, particularly among older populations. In contrast, low-SDI regions show less pronounced reductions or even stagnation in smoking-attributable IHD rates. Many middle- and low-SDI regions continue to struggle with high smoking prevalence, limited healthcare resources, and insufficient tobacco control measures. Regions such as Eastern Europe, Central Asia, and parts of Southeast Asia show slower progress, underscoring the challenges in implementing comprehensive smoking regulations and public health initiatives where economic constraints and cultural factors pose significant barriers.

Gender disparities are evident, with males consistently bearing a higher burden of smoking-attributable IHD across all SDI levels, consistent with the higher prevalence of smoking among men^24,25^. This disparity is most pronounced in middle-SDI regions, where male smoking rates are significantly higher than female rates, contributing to elevated ASDRs and ASRDALYs. While targeted public health policies in high-SDI countries have narrowed the gender gap, males in low-SDI regions continue to face substantial risks. Cultural norms and limited access to cessation support further exacerbate this disparity, reinforcing the need for gender-specific interventions. Although the smoking-related IHD burden is much lower in females, the death rate and DALYs increase sharply in older age groups, potentially due to factors such as education, economic status, and social isolation^26-29^.

Age-specific analysis indicates that older smokers, both male and female, bear the highest burden of smoking-related IHD, as aging inherently increases IHD risk^30,31^, and the cumulative effects of smoking worsen over time^32^. Over the past 30 years, older age groups (≥75 years) have seen the greatest declines in smoking-related IHD burden, particularly in high-SDI regions. This trend reflects the success of long-term anti-smoking measures and advancements in cardiovascular care for older adults. However, in low-SDI regions, the decline among older populations has been minimal or, in some cases, has reversed, highlighting the need for targeted interventions to address these disparities.

These trends emphasize the importance of sustained, region-specific tobacco control strategies to reduce the IHD burden from smoking^33^. High-SDI regions can serve as models, demonstrating the benefits of consistent anti-smoking policies, accessible healthcare, and ongoing preventive care^34^. However, middle- and low-SDI regions face challenges that require coordinated global efforts to strengthen anti-smoking measures, improve healthcare access, and address socio-economic barriers to smoking cessation^34^.

Gender disparities also require tailored approaches to address the health risks faced by both males and females. In high-prevalence regions, males continue to bear a significant burden of smoking-related IHD, while rising smoking rates among females in some middle-SDI countries also pose an increasing risk. As female smoking rates rise in some middle-SDI countries, proactive interventions are needed to prevent an increase in IHD burden among women^28,35^.

Based on the findings of the referenced study, global smoking prevalence and the associated loss of life are projected to continue improving in the future^8^. This aligns with the global trends observed in our study over the past three decades. However, it is noteworthy that in the last 30 years, progress in reducing smoking-attributable burdens has been slow or even reversed in low-income regions, particularly among older adults and male populations. Without enhanced policy interventions and healthcare support targeting these vulnerable groups, they may fail to benefit from the future global improvements in smoking prevalence and the associated health outcomes, thus continuing to bear significant health losses due to smoking.

### Limitations

This study has several limitations that should be acknowledged. First, the accuracy of the estimates depends on the quality and availability of data used in the GBD 2021 study, which may vary significantly across regions. Data gaps in certain low- and middle-SDI regions may have introduced biases, potentially leading to underestimation or overestimation of the true IHD burden attributable to smoking. Second, this study relied on EAPCs to assess temporal trends. These estimates are derived from statistical models and may be influenced by assumptions made during modeling, shifts in data quality, or changes in coding practices over time. Third, as this is a secondary analysis of GBD 2021 data, which are modeled estimates incorporating adjustments for multiple covariates and subgroup stratifications, the results are primarily descriptive. Therefore, statistical comparisons between subgroups are not feasible, and findings should be interpreted with caution. Finally, the analysis did not account for secondhand smoking or the emerging burden of e-smoking, which may contribute to IHD burden and require further exploration in future studies. Future research should focus on improving data collection in underserved regions, addressing residual confounding, and incorporating additional smoking-related behaviors to provide a more comprehensive understanding of IHD burden trends.

## CONCLUSIONS

This study demonstrates notable global reductions in the burden of IHD attributable to smoking, primarily led by high-SDI regions with effective public health measures. However, disparities remain, particularly in lower-SDI regions, males and older populations (aged ≥75 years), who face higher burdens due to elevated smoking rates and limited healthcare access. Addressing these inequalities requires targeted, region-specific interventions to further reduce the impact of smoking-related IHD. Strengthening tobacco control, expanding healthcare access, and implementing gender-specific strategies are crucial for achieving equitable improvements in cardiovascular health worldwide.

## Supplementary Material



## Data Availability

The list of data sources used is publicly available on the Global Health Data Exchange (GHDx) platform (https://vizhub.healthdata.org/gbd-results/).
