# Supplementary material for: Global trends in the burden of ischemic heart disease attributable to smoking from 1990 to 2021: A systematic analysis of the Global Burden of Disease Study 2021
Source: Tob Induc Dis. 2025 Jan 29;23:10.18332/tid/199931. doi: 10.18332/tid/199931 (PMC11775718; doi:10.18332/tid/199931)
Supplement: Supplementary file 1 [file TID-23-07-s1.pdf]

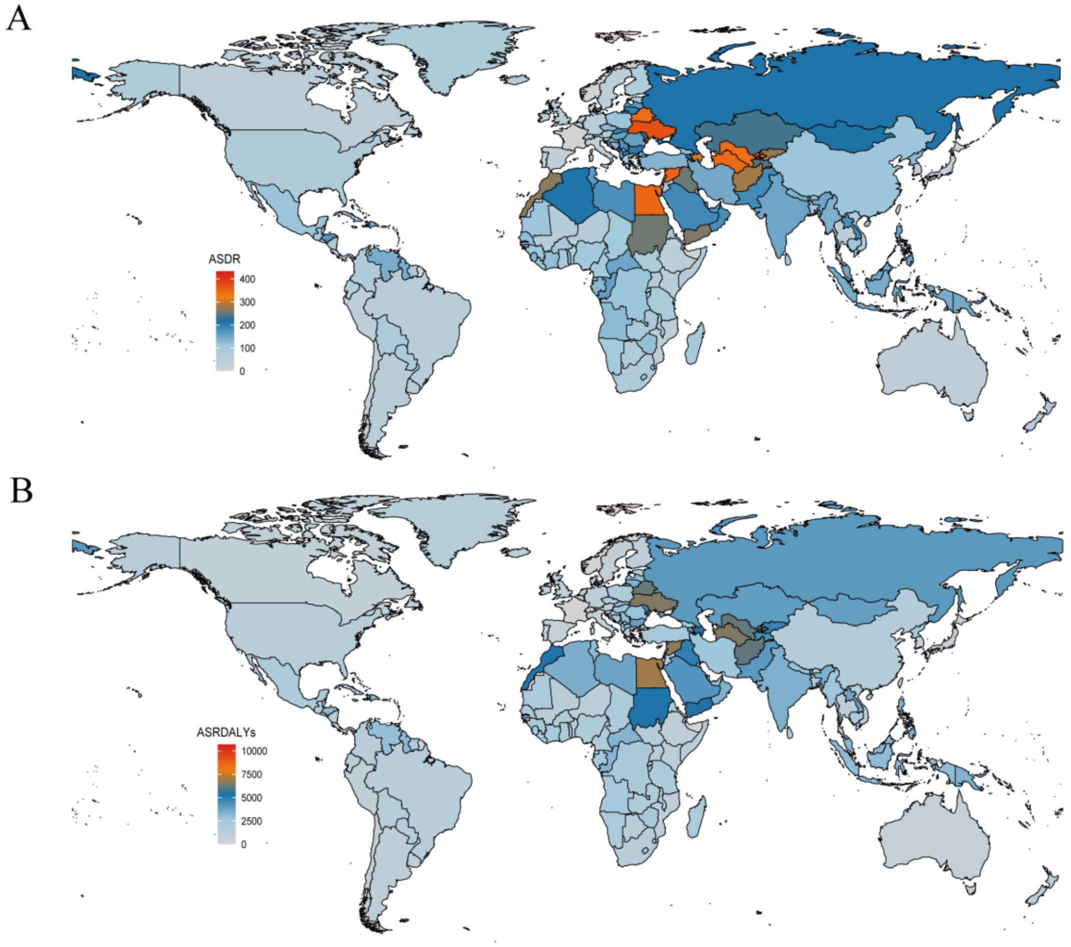

Supplementary Figure 1. Global distribution of IHD burden among 204 countries and territories in 2021

Global distribution of age-standardized death rates (ASDR, A) and age-standardized disability-adjusted life years (ASRDALYs, B) per 100,000 population for ischemic heart disease (IHD) in 2021. Warm colors indicate regions with a higher IHD burden, while cool colors representing regions represent regions with a lower burden. Data are presented for both sexes across global regions.

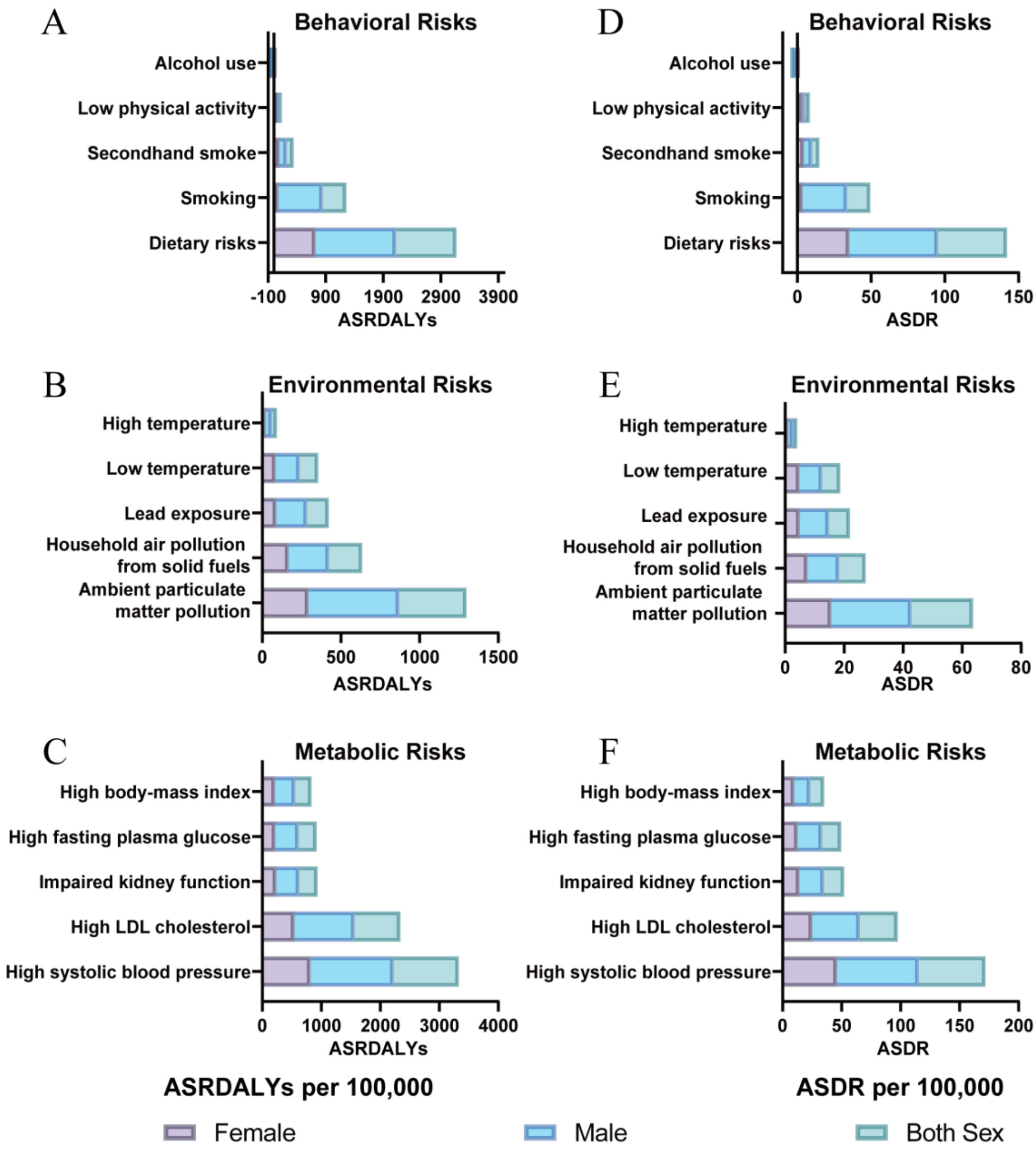

Supplementary Figure 2. Leading risk factors for IHD by risk category and sex in 2021

The top behavioral, environmental, and metabolic risk factors contributing to IHD burden in 2021 are illustrated. ASRDALYs for behavioral risks (A), environmental risks (B), and metabolic risks (C) and ASDR for behavioral risks (D), environmental risks (E), and metabolic risks (F) are shown. Data are stratified by sex (both sexes, females, and males).

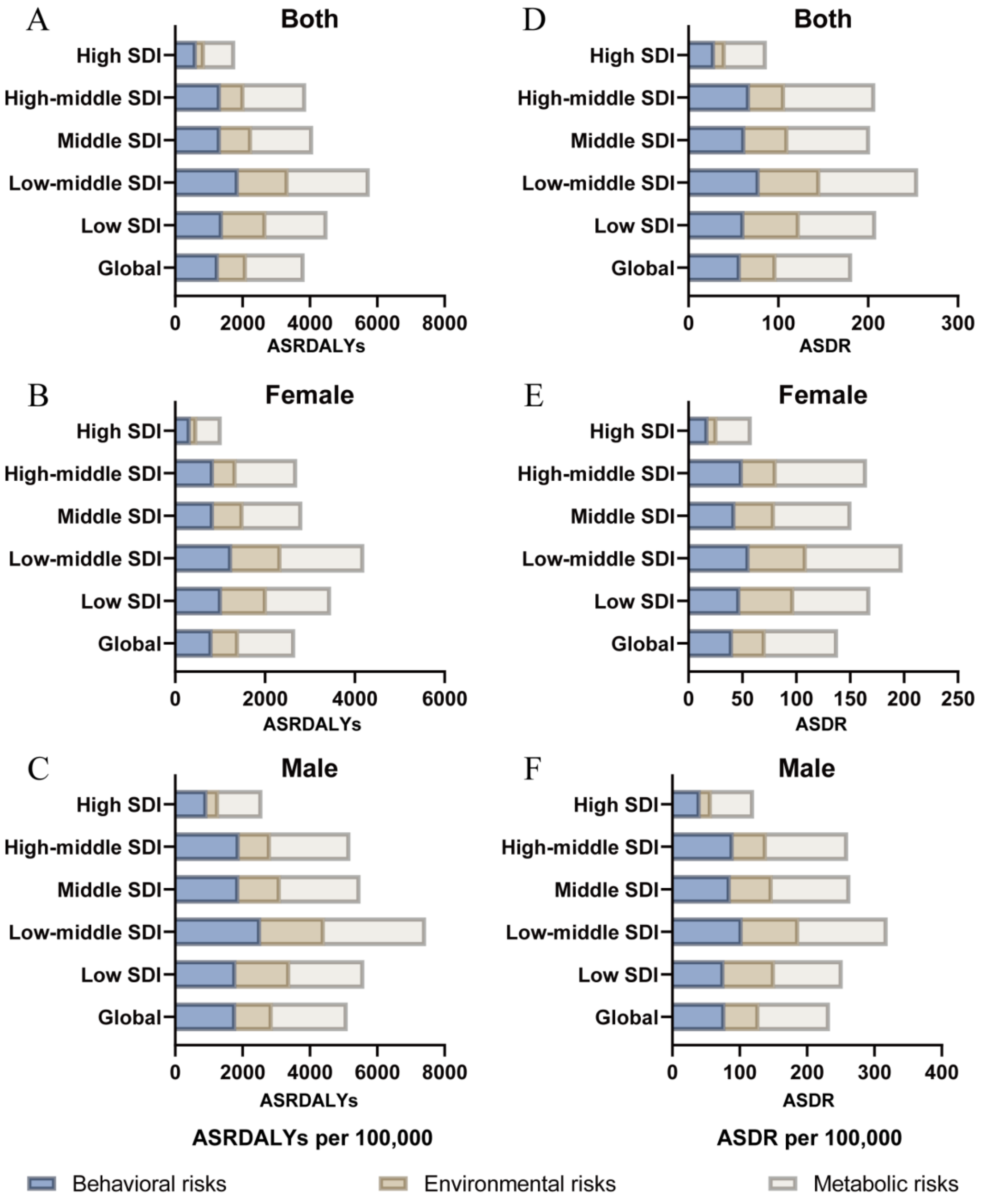

Supplementary Figure 3. Distribution of risk factors for IHD by SDI level and sex in 2021

The contribution of behavioral, environmental, and metabolic risk factors to IHD burden across socio-demographic index (SDI) regions is illustrated. ASRDALYs for both sexes (A), females (B), and males (C), and ASDR for both sexes (D), females (E), and males (F) are shown. Data are stratified by SDI regions.

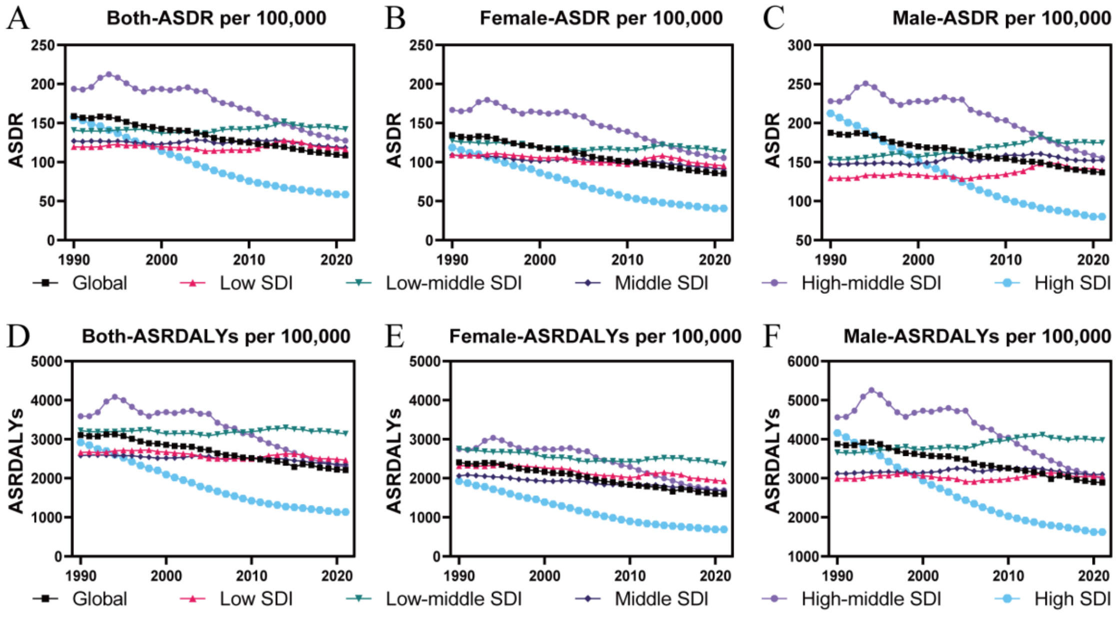

Supplementary Figure 4. Temporal trends in IHD burden

The temporal trends in the IHD burden from 1990 to 2021, represented by ASDR per 100,000 population for both sexes (A), females (B), and males (C), and by ASRDALYs per 100,000 population for both sexes (D), females (E), and males (F), stratified by socio-demographic index (SDI) levels.

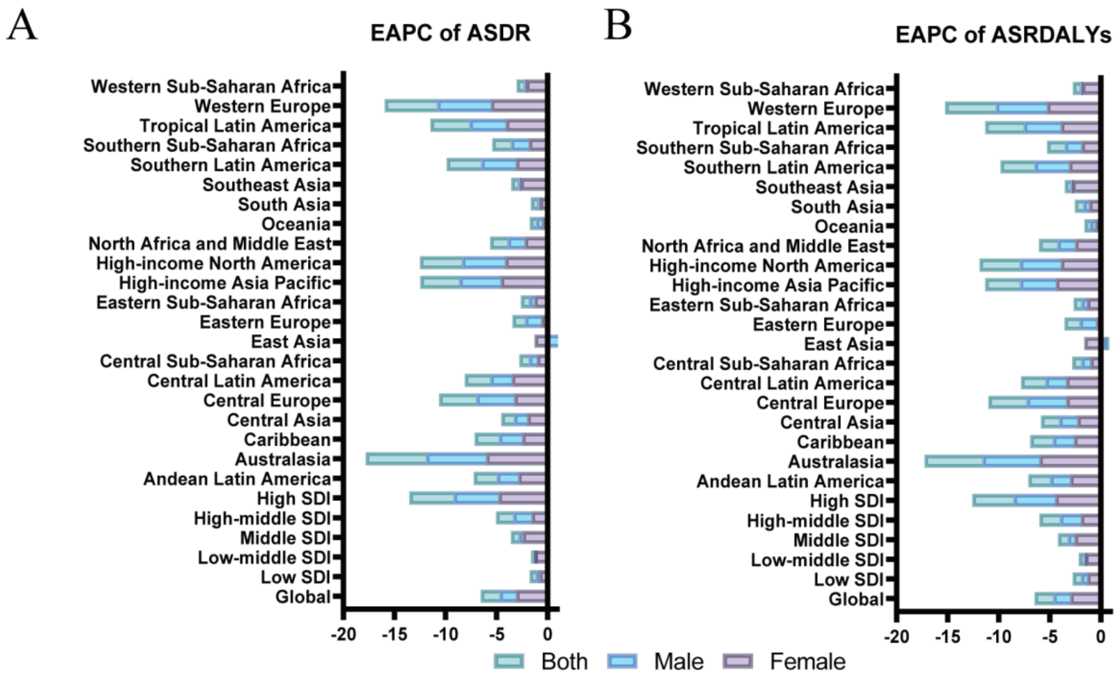

Supplementary Figure 5. Trends in the EAPC of IHD burden attributable to smoking from 1990 to 2021

The estimated annual percentage changes (EAPC) of smoking-attributable IHD burden are illustrated for ASDR (A) and ASRDALYs (B) from 1990 to 2021. The data are stratified by global,

SDI levels, and specific geographic regions. Bars represent EAPC values for both sexes, males, and females.

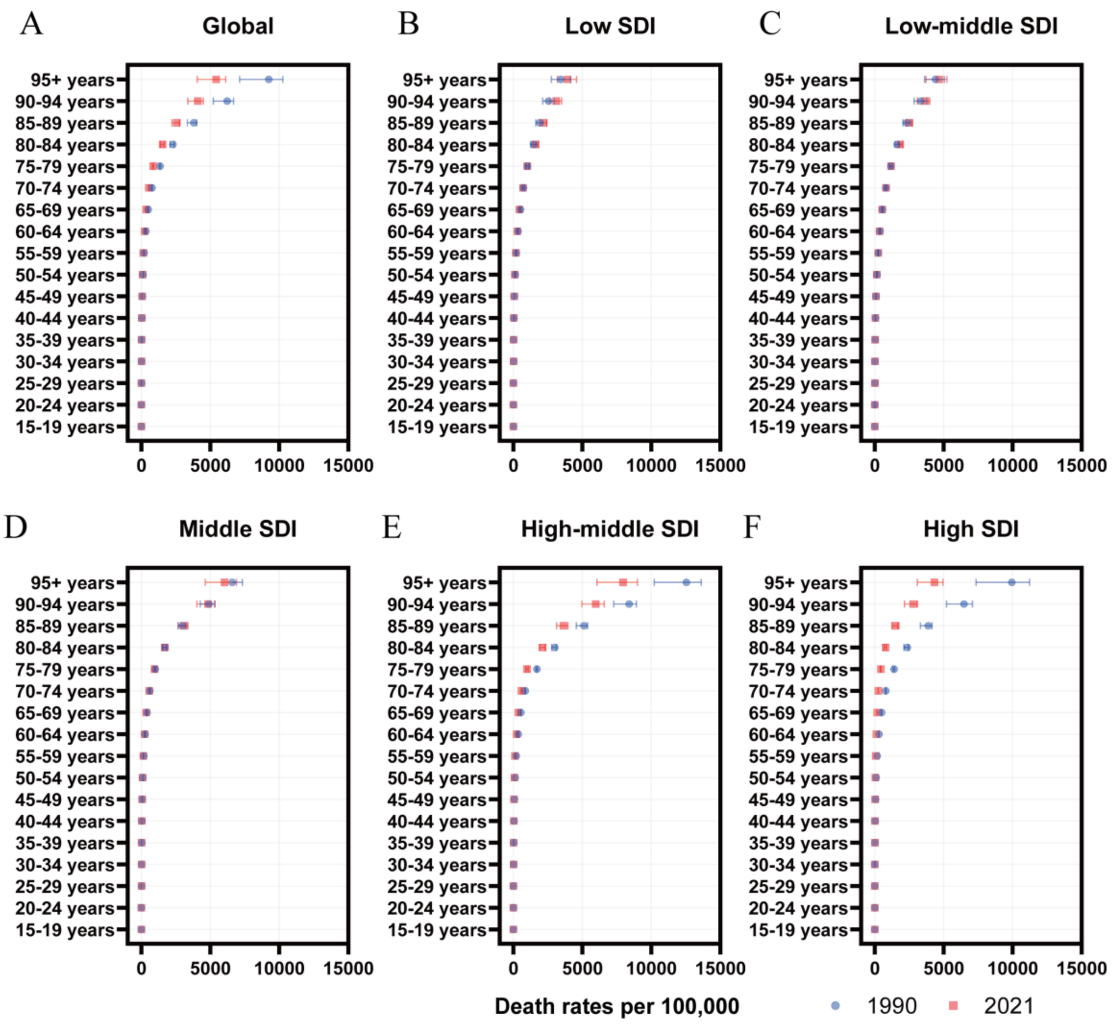

Supplementary Figure 6. Death rates for IHD by age groups across global and SDI levels in 1990 and 2021

The death rates (per 100,000 population) for IHD are shown for 1990 (blue) and 2021 (red) across different age groups, stratified by global regions (A), low SDI (B), low-middle SDI (C), middle SDI (D), high-middle SDI (E), and high SDI (F). Error bars indicate the 95% uncertainty intervals (UI).

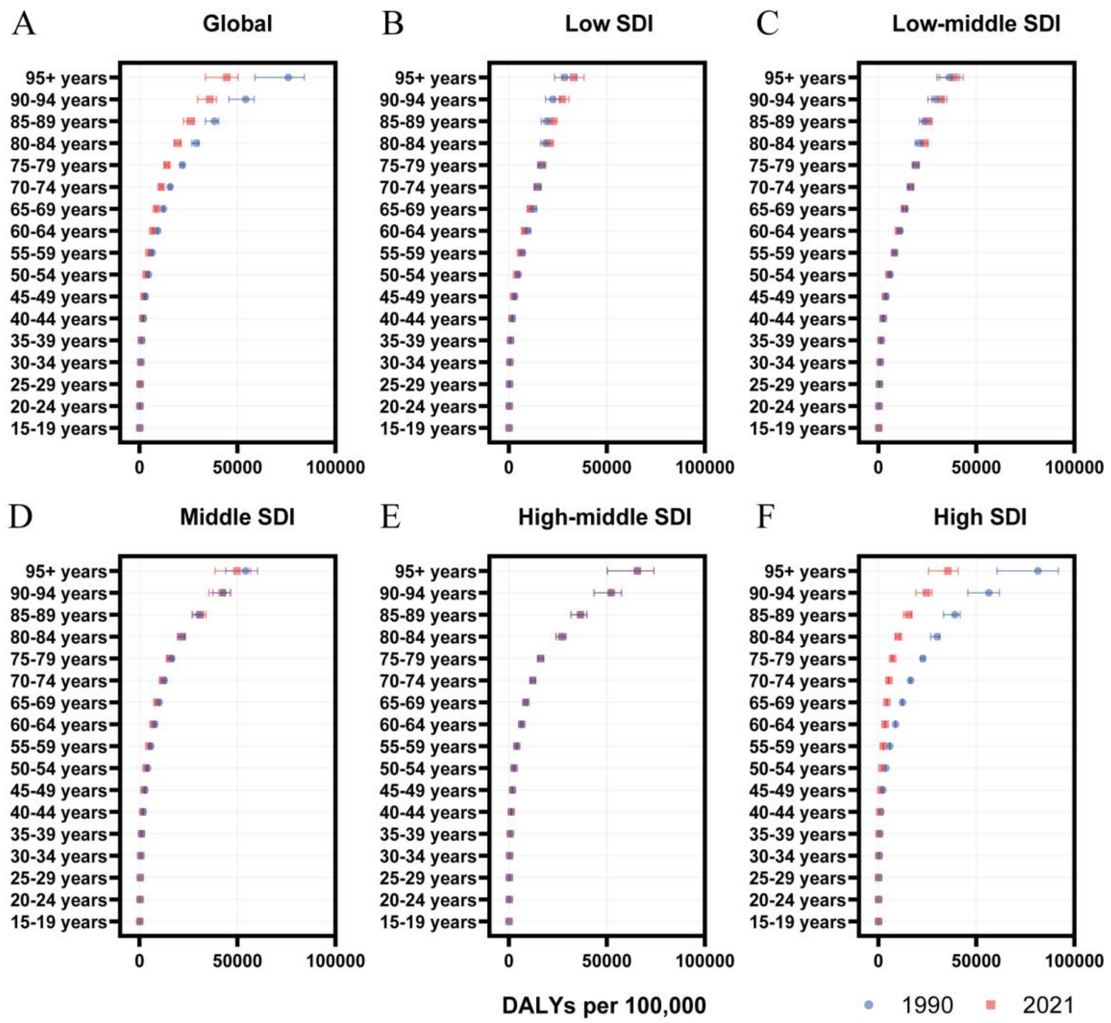

Supplementary Figure 7. DALYs for IHD by age groups across global and SDI level in 1990 and 2021

The DALYs (disability-adjusted life years, per 100,000 populations) for IHD are shown for 1990 (blue) and 2021 (red) across different age groups, stratified by global regions (A), low SDI (B), low-middle SDI (C), middle SDI (D), high-middle SDI (E), and high SDI (F). Error bars indicate the 95% UI.

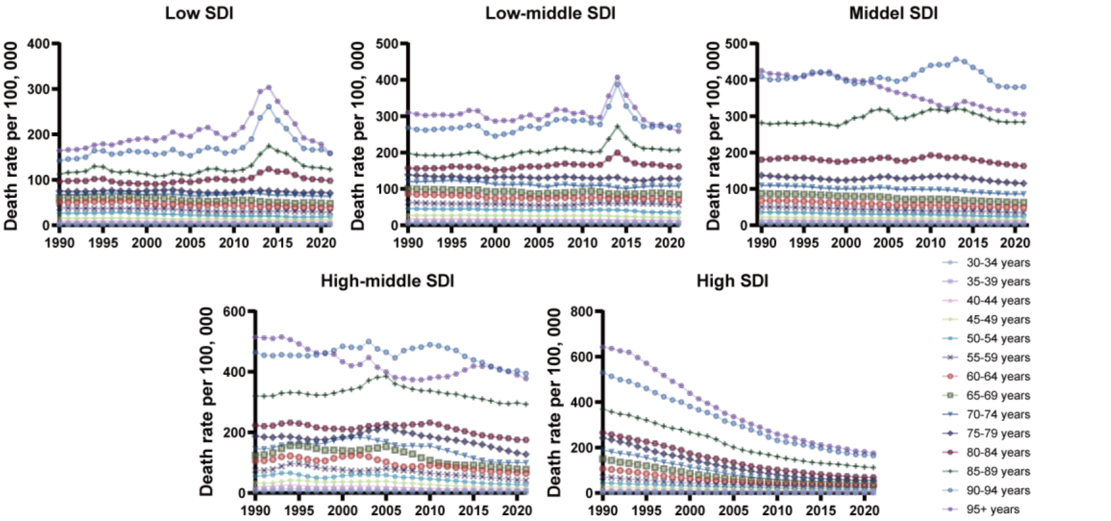

Figure 8. Temporal trends in death rates for IHD burden attributable to smoking across age groups and SDI regions

Temporal trends in death rates per 100,000 population for IHD attributable to smoking from 1990 to 2021 across different age groups are presented, stratified by SDI regions: low SDI, low-middle SDI, middle SDI, high-middle SDI, and high SDI.

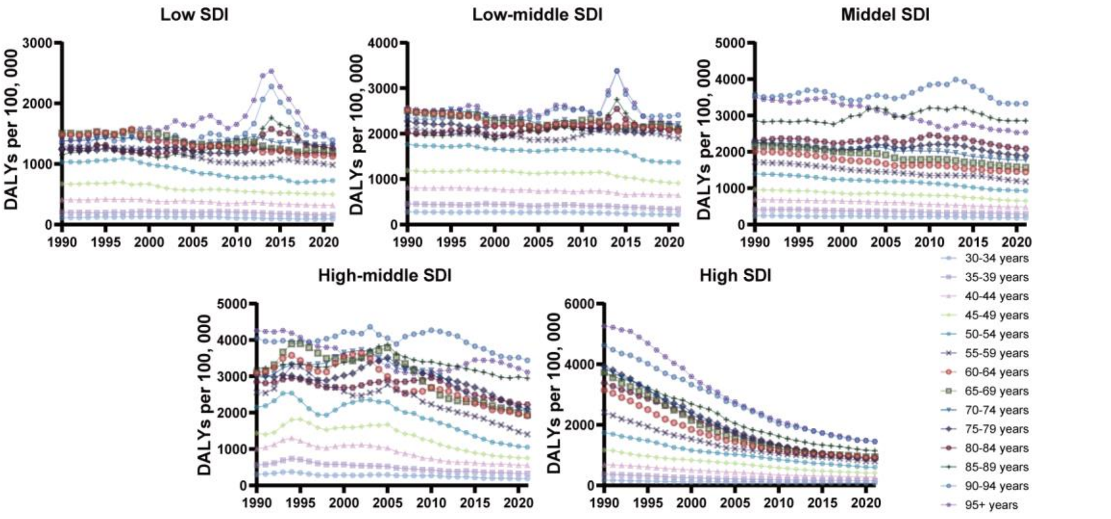

Supplementary Figure 9. Temporal trends in DALYs for IHD burden attributable to smoking across age groups and SDI regions

Temporal trends in DALYs per 100,000 population for IHD attributable to smoking from 1990 to 2021 across different age groups are presented, stratified by SDI regions: low SDI, low-middle SDI, middle SDI, high-middle SDI, and high SDI.

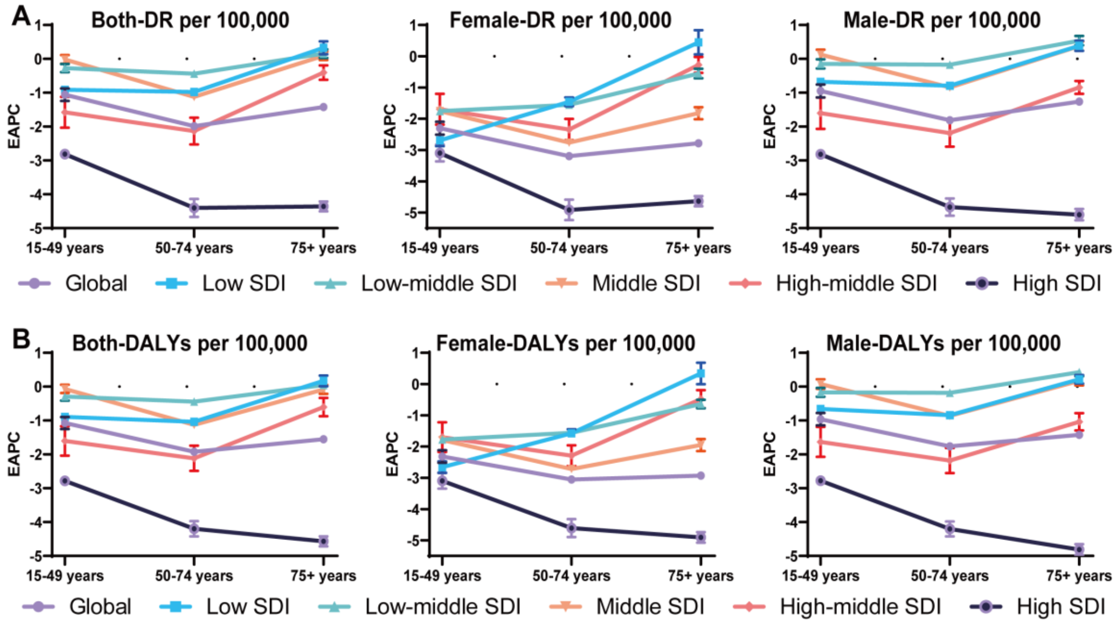

Supplementary Figure 10. Trends in the EAPC of IHD burden attributable to smoking from 1990 to 2021

The EAPC of smoking-attributable IHD burden of DR (A) and DALYs (B) from 1990 to 2021 were calculated for both sexes, females, and males, stratified by age groups (15-49 years, 50-74 years, and 75+ years). Error bars indicate the 95% CI.

Location	ASDR (95% UI)		ASRDALYs (95% UI)		EAPC (95% CI)	
	1990	2021	1990	2021	ASDR	ASRDALYs
Global	158.90 (148.14, 165.30)	108.73 (99.60, 115.38)	3107.61 (2966.50, 3222.67)	2212.16 (2075.54, 2327.61)	-1.22 (-0.79, -1.64)	-1.09 (-0.71, -1.47)
**SDI**						
Low SDI	119.50 (106.26, 132.45)	116.41 (105.21, 127.69)	2668.94 (2235.87, 2725.20)	2464.12 (2361.30, 2967.21)	-0.08 (-0.05, -0.11)	-0.26 (-0.17, -0.35)
Low-middle SDI	140.99 (131.30, 151.87)	142.10 (129.76, 151.24)	3221.76 (2912.65, 3360.62)	3138.58 (2993.33, 3437.47)	0.03 (0.02, 0.03)	-0.08 (-0.05, -0.11)
Middle SDI	127.06 (107.23, 127.80)	118.71 (118.35, 134.97)	2593.46 (2447.25, 2745.56)	2351.21 (2174.84, 2514.63)	-0.22 (-0.14, -0.30)	-0.32 (-0.20, -0.43)
High-middle SDI	193.94 (182.11, 200.60)	127.50 (115.03, 137.72)	3589.76 (2122.97, 2482.46)	2301.49 (3435.61, 3712.83)	-1.34 (-0.87, -1.82)	-1.42 (-0.92, -1.92)
High SDI	157.59 (52.18, 61.92)	58.45 (144.19, 163.92)	2919.50 (1053.56, 1186.53)	1134.02 (2762.75, 3002.02)	-3.15 (-2.04, -4.26)	-3.00 (-1.95, -4.06)
**Region**						
Andean Latin America	92.95 (84.56, 101.24)	58.17 (49.56, 69.33)	1858.09 (976.72, 1369.62)	1150.61 (1696.99, 2045.72)	-1.50 (-0.97, -2.03)	-1.53 (-0.99, -2.07)
Australasia	176.76 (162.82, 184.02)	46.67 (40.23, 50.21)	3237.02 (3058.20, 3338.90)	812.69 (739.29, 858.37)	-4.20 (-2.72, -5.68)	-4.36 (-2.83, -5.90)
Caribbean	188.97 (179.08, 195.79)	112.50 (100.51, 126.54)	3723.19 (2112.34, 2730.37)	2397.80 (3551.61, 3873.17)	-1.66 (-1.07, -2.24)	-1.41 (-0.91, -1.91)
Central Asia	320.47 (299.83, 332.36)	265.51 (240.67, 290.42)	6206.85 (5937.37, 6407.43)	4864.49 (4415.55, 5338.75)	-0.61 (-0.39, -0.82)	-0.78 (-0.51, -1.06)
Central Europe	272.56 (259.22, 279.18)	139.98 (126.84, 148.91)	5198.33 (5046.13, 5293.63)	2471.23 (2288.20, 2635.38)	-2.13 (-1.38, -2.88)	-2.37 (-1.54, -3.20)
Central Latin America	125.72 (92.48, 114.59)	103.69 (118.69, 129.14)	2445.66 (2357.94, 2497.28)	2016.97 (1821.53, 2238.28)	-0.62 (-0.40, -0.84)	-0.62 (-0.40, -0.84)
Central Sub-Saharan Africa	134.90 (107.86, 167.20)	119.34 (93.70, 150.29)	2816.50 (2225.44, 3535.98)	2433.06 (1902.53, 3100.83)	-0.39 (-0.26, -0.53)	-0.47 (-0.31, -0.64)
East Asia	93.92 (83.87, 105.30)	108.90 (91.18, 125.79)	1770.78 (1574.00, 1979.40)	1839.92 (1541.21, 2135.32)	0.48 (0.31, 0.65)	0.12 (0.08, 0.17)
Eastern Europe	323.17 (305.26, 331.76)	252.89 (226.96, 277.15)	5957.77 (5735.98, 6088.42)	4687.70 (4267.79, 5115.68)	-0.79 (-0.51, -1.07)	-0.77 (-0.50, -1.04)
Eastern Sub-Saharan Africa	69.44 (61.05, 78.85)	72.16 (62.09, 82.99)	1556.44 (1333.12, 1773.89)	1536.64 (1388.67, 1773.02)	0.12 (0.08, 0.17)	-0.04 (-0.03, -0.06)
High-income Asia Pacific	67.04 (59.85, 70.50)	25.56 (21.78, 27.65)	1165.81 (1080.72, 1212.46)	492.99 (446.93, 520.22)	-3.06 (-1.98, -4.14)	-2.74 (-1.77, -3.70)
High-income North America	177.72 (160.27, 186.07)	75.85 (67.17, 80.60)	3347.44 (3141.10, 3449.54)	1461.90 (1350.51, 1526.21)	-2.71 (-1.76, -3.66)	-2.64 (-1.71, -3.57)
North Africa and Middle East	275.18 (180.59, 223.68)	202.85 (253.62, 299.12)	5763.47 (5366.03, 6251.52)	4023.22 (3581.71, 4507.47)	-0.98 (-0.63, -1.32)	-1.15 (-0.75, -1.56)
Oceania	182.55 (155.47, 217.42)	170.89 (145.43, 201.15)	4259.05 (3323.16, 4723.71)	3962.77 (3546.41, 5124.64)	-0.21 (-0.14, -0.29)	-0.23 (-0.15, -0.31)
South Asia	136.39 (122.87, 149.55)	149.14 (136.97, 161.16)	3276.85 (2983.93, 3564.63)	3351.09 (3075.41, 3616.42)	0.29 (0.19, 0.39)	0.07 (0.05, 0.10)
Southeast Asia	114.72 (103.39, 125.65)	110.92 (100.18, 120.20)	2549.17 (2315.76, 2761.72)	2415.55 (2177.87, 2635.30)	-0.11 (-0.07, -0.15)	-0.17 (-0.11, -0.23)

Southern Latin America	149.43 (50.08, 57.26)	54.41 (141.12, 154.54)	2821.47 (1012.57, 1113.22)	1070.86 (2718.03, 2901.05)	-3.21 (-2.08, -4.34)	-3.08 (-1.99, -4.16)
Southern Sub-Saharan Africa	75.96 (76.93, 90.19)	83.44 (64.95, 84.84)	1626.44 (1424.74, 1789.71)	1689.53 (1565.07, 1832.24)	0.30 (0.20, 0.41)	0.12 (0.08, 0.17)
Tropical Latin America	135.91 (126.39, 140.82)	64.49 (58.98, 67.84)	2899.87 (2774.08, 2979.35)	1476.14 (1385.48, 1533.17)	-2.38 (-1.54, -3.21)	-2.15 (-1.40, -2.91)
Western Europe	148.22 (41.45, 50.42)	47.27 (136.54, 153.85)	2740.65 (775.27, 886.10)	843.77 (2604.66, 2812.94)	-3.62 (-2.35, -4.89)	-3.73 (-2.42, -5.04)
Western Sub-Saharan Africa	105.29 (90.24, 121.56)	105.97 (92.83, 120.17)	2082.33 (1785.44, 2418.30)	2029.03 (1760.44, 2334.06)	0.02 (0.01, 0.03)	-0.08 (-0.05, -0.11)

Supplementary Table 1. Global Distribution and EAPC of IHD-Related ASDR and ASRDALYs from 1990 to 2021

The age-standardized death rates (ASDR) and age-standardized disability-adjusted life years (ASRDALYs) per 100,000 population of ischemic heart disease (IHD) among both sexes across global, SDI levels, and specific geographic regions for the years 1990 and 2021 are summarized. Temporal trends from 1990 to 2021 are represented by the estimated annual percentage change (EAPC) of ASDR and ASRDALYs, with corresponding 95% confidence intervals (CI). Positive EAPC values indicate an increasing trend, while negative EAPC values denote a decreasing trend.

Location	ASDR (95% UI)		ASRDALYs (95% UI)		EAPC (95% CI)	
	1990	2021	1990	2021	ASDR	ASDR
Global	8.98 (7.38, 10.77)	3.54 (2.83, 4.32)	198.32 (167.06, 232.83)	80.19 (66.48, 95.81)	-3.10 (-3.64, -2.56)	-3.02 (-3.51, -2.53)
**SDI**						
Low SDI	4.80 (3.64, 6.13)	3.42 (2.55, 4.48)	122.51 (93.69, 153.45)	79.12 (60.53, 102.09)	-0.85 (-2.85, 1.19)	-1.36 (-2.67, -0.04)
Low-middle SDI	5.84 (4.64, 7.26)	3.70 (2.88, 4.69)	149.38 (120.63, 182.88)	90.25 (71.73, 112.76)	-1.31 (-2.10, -0.51)	-1.53 (-2.35, -0.71)
Middle SDI	5.96 (4.79, 7.29)	2.87 (2.24, 3.63)	135.46 (112.57, 161.34)	63.54 (51.56, 78.52)	-2.47 (-3.53, -1.41)	-2.58 (-3.18, -1.98)
High-middle SDI	7.04 (5.75, 8.50)	4.37 (3.30, 5.66)	168.41 (142.65, 200.71)	94.69 (76.68, 116.58)	-1.60 (-4.50, 1.39)	-1.98 (-5.00, 1.15)
High SDI	13.95 (11.42, 16.77)	3.41 (2.76, 4.15)	306.21 (259.82, 357.55)	81.48 (68.50, 96.13)	-4.82 (-6.15, -3.47)	-4.48 (-5.79, -3.15)
**Region**						
Andean Latin America	2.43 (1.79, 3.06)	1.12 (0.81, 1.50)	59.64 (45.70, 74.89)	26.09 (19.54, 34.54)	-2.89 (-6.87, 1.26)	-3.03 (-6.83, 0.91)
Australasia	13.74 (11.11, 16.61)	2.18 (1.65, 2.79)	301.88 (251.67, 354.84)	47.90 (38.75, 58.18)	-6.03 (-6.81, -5.24)	-6.04 (-7.31, -4.76)
Caribbean	13.13 (10.49, 16.11)	6.14 (4.62, 7.90)	330.93 (272.03, 398.14)	150.29 (116.05, 190.12)	-2.51 (-3.53, -1.49)	-2.64 (-3.86, -1.41)
Central Asia	3.27 (2.68, 4.00)	2.11 (1.67, 2.60)	92.05 (76.08, 110.57)	55.95 (44.68, 68.61)	-2.01 (-6.27, 2.44)	-2.32 (-7.00, 2.61)
Central Europe	15.87 (13.15, 18.70)	6.36 (5.10, 7.68)	406.44 (346.00, 466.30)	157.68 (130.28, 185.25)	-3.28 (-4.36, -2.18)	-3.40 (-4.43, -2.36)
Central Latin America	6.87 (5.38, 8.61)	2.62 (2.07, 3.29)	162.43 (133.73, 193.41)	64.22 (50.82, 79.66)	-3.53 (-5.41, -1.61)	-3.41 (-5.49, -1.28)
Central Sub-Saharan Africa	1.22 (0.81, 1.70)	0.91 (0.61, 1.29)	31.82 (21.33, 45.57)	23.80 (15.95, 33.53)	-1.07 (-2.40, 0.28)	-1.05 (-2.15, 0.06)
East Asia	6.26 (4.58, 8.47)	3.97 (2.58, 5.71)	121.82 (92.59, 158.25)	69.24 (47.58, 95.83)	-1.29 (-4.41, 1.94)	-1.64 (-4.23, 1.01)
Eastern Europe	4.68 (3.67, 5.74)	4.24 (3.40, 5.28)	125.14 (101.45, 149.56)	123.74 (101.51, 151.82)	-0.65 (-8.11, 7.41)	-0.48 (-8.27, 7.98)
Eastern Sub-Saharan Africa	1.98 (1.48, 2.66)	1.48 (1.05, 2.01)	45.30 (34.32, 59.31)	32.96 (24.37, 43.56)	-1.33 (-2.49, -0.14)	-1.39 (-2.55, -0.23)
High-income Asia Pacific	2.85 (2.20, 3.69)	0.68 (0.50, 0.92)	60.14 (48.67, 73.12)	15.51 (12.32, 19.52)	-4.61 (-5.30, -3.91)	-4.41 (-5.22, -3.59)
High-income North America	19.72 (16.18, 23.84)	6.12 (4.88, 7.45)	450.63 (383.11, 526.56)	147.46 (122.24, 173.48)	-4.17 (-5.78, -2.53)	-3.92 (-5.46, -2.37)
North Africa and Middle East	7.66 (6.00, 9.74)	4.06 (3.09, 5.17)	196.96 (158.60, 244.47)	97.34 (75.13, 122.12)	-2.26 (-3.11, -1.41)	-2.54 (-3.35, -1.72)
Oceania	11.98 (8.66, 16.36)	10.34 (7.68, 13.77)	369.54 (262.38, 510.44)	319.66 (236.53, 426.74)	-0.57 (-1.91, 0.79)	-0.57 (-2.00, 0.89)
South Asia	5.44 (4.19, 7.02)	3.86 (2.87, 5.10)	141.92 (111.35, 181.02)	92.55 (70.74, 120.92)	-0.79 (-2.44, 0.90)	-1.17 (-2.53, 0.21)
Southeast Asia	6.83 (5.41, 8.58)	3.22 (2.48, 4.10)	160.97 (128.10, 196.16)	72.70 (57.63, 90.44)	-2.69 (-4.26, -1.10)	-2.81 (-4.13, -1.46)

Southern Latin America	9.31 (7.48, 11.35)	3.15 (2.60, 3.86)	245.65 (204.67, 290.03)	85.12 (71.90, 100.87)	-3.13 (-4.20, -2.05)	-3.13 (-4.24, -2.01)
Southern Sub-Saharan Africa	5.44 (4.13, 7.09)	3.06 (2.34, 3.99)	136.72 (107.84, 168.95)	72.72 (56.62, 90.55)	-1.86 (-4.73, 1.10)	-1.91 (-4.44, 0.68)
Tropical Latin America	16.50 (12.90, 20.74)	4.96 (3.97, 6.16)	409.72 (334.37, 486.76)	133.35 (109.28, 163.24)	-4.12 (-6.03, -2.18)	-3.92 (-5.45, -2.37)
Western Europe	12.14 (9.97, 14.52)	2.28 (1.84, 2.76)	262.23 (221.96, 305.43)	52.97 (44.49, 62.19)	-5.58 (-6.63, -4.52)	-5.28 (-6.40, -4.15)
Western Sub-Saharan Africa	1.75 (1.23, 2.51)	0.95 (0.69, 1.30)	38.41 (28.38, 52.61)	22.41 (16.61, 29.52)	-2.13 (-3.57, -0.67)	-1.87 (-3.38, -0.33)

Supplementary Table 2. Global Distribution and EAPC of IHD burden attributable to smoking (female)

The ASDR and ASRDALYs per 100,000 population of IHD attributable to smoking among females across global regions, SDI levels, and specific geographic regions for 1990 and 2021 are summarized. Temporal trends from 1990 to 2021 are represented by the EAPC of ASDR and ASRDALYs, with corresponding 95% CI. Positive EAPC values indicate an increasing trend, while negative EAPC values denote a decreasing trend

Location	ASDR (95% UI)		ASRDALYs (95% UI)		EAPC (95% CI)	
	1990	2021	1990	2021	ASDR	ASRDALYs
Global	49.31 (42.15, 56.81)	30.24 (25.38, 35.58)	1244.21 (1090.11, 1401.08)	768.01 (661.43, 884.19)	-1.64 (-2.14, -1.13)	-1.66 (-2.31, -1.00)
**SDI**						
Low SDI	21.90 (17.87, 26.32)	19.66 (15.87, 23.94)	604.45 (493.80, 728.51)	516.83 (424.73, 619.79)	-0.37 (-1.11, 0.38)	-0.61 (-1.30, 0.08)
Low-middle SDI	40.78 (33.68, 48.14)	38.88 (32.47, 45.70)	1131.13 (962.40, 1323.40)	1027.03 (866.63, 1190.30)	-0.01 (-0.73, 0.72)	-0.18 (-0.83, 0.48)
Middle SDI	39.66 (33.21, 46.27)	33.69 (27.70, 40.26)	1013.52 (876.56, 1159.49)	806.91 (676.10, 939.35)	-0.40 (-1.17, 0.37)	-0.67 (-1.26, -0.07)
High-middle SDI	62.72 (54.01, 71.76)	38.77 (32.00, 46.39)	1586.82 (1399.28, 1772.25)	937.11 (794.58, 1089.07)	-1.79 (-4.54, 1.04)	-2.05 (-5.05, 1.04)
High SDI	56.71 (48.54, 65.38)	15.46 (12.85, 18.11)	1372.08 (1202.73, 1547.92)	406.53 (347.45, 469.86)	-4.41 (-5.91, -2.89)	-4.09 (-5.55, -2.61)
**Region**						
Andean Latin America	15.04 (12.01, 17.84)	8.35 (6.28, 10.73)	381.91 (316.71, 449.01)	220.16 (167.86, 279.67)	-5.90 (-7.47, -4.31)	-5.56 (-7.16, -3.94)
Australasia	41.73 (35.18, 49.09)	7.02 (5.79, 8.50)	1066.15 (916.68, 1231.76)	195.03 (165.48, 229.58)	-2.28 (-3.97, -0.57)	-2.07 (-3.82, -0.30)
Caribbean	43.49 (36.51, 51.65)	22.44 (17.80, 27.40)	1106.33 (955.95, 1270.82)	602.74 (490.21, 731.19)	-1.30 (-4.64, 2.16)	-1.78 (-5.48, 2.06)
Central Asia	98.25 (83.71, 114.00)	71.91 (59.99, 84.70)	2559.89 (2240.78, 2895.60)	1687.46 (1405.14, 1962.17)	-3.74 (-4.83, -2.64)	-3.87 (-4.96, -2.76)
Central Europe	98.14 (83.95, 112.31)	34.38 (28.52, 40.62)	2590.25 (2288.41, 2882.56)	873.39 (739.63, 1012.13)	-2.11 (-3.61, -0.59)	-2.04 (-3.74, -0.31)
Central Latin America	26.40 (22.33, 30.64)	15.21 (12.05, 18.35)	672.45 (580.77, 764.05)	398.02 (322.73, 473.46)	-0.80 (-3.05, 1.50)	-0.88 (-3.07, 1.35)
Central Sub-Saharan Africa	18.37 (13.34, 24.24)	14.93 (11.05, 19.72)	520.53 (380.02, 693.66)	411.84 (296.90, 547.19)	1.01 (-1.55, 3.64)	0.58 (-1.38, 2.57)
East Asia	33.60 (26.92, 41.08)	38.98 (29.39, 50.72)	777.72 (627.35, 937.57)	812.74 (626.37, 1030.63)	-1.58 (-7.64, 4.86)	-1.64 (-7.96, 5.11)
Eastern Europe	118.07 (102.27, 135.18)	84.92 (69.59, 101.13)	2989.91 (2650.01, 3351.01)	2179.23 (1827.00, 2547.81)	-0.53 (-1.44, 0.40)	-0.54 (-1.42, 0.35)
Eastern Sub-Saharan Africa	11.10 (8.90, 13.68)	10.26 (7.90, 12.70)	307.01 (249.36, 373.69)	282.58 (222.04, 348.04)	-4.07 (-4.76, -3.37)	-3.54 (-4.17, -2.90)
High-income Asia Pacific	26.11 (21.87, 30.31)	7.64 (6.42, 8.98)	582.26 (506.41, 654.02)	200.51 (171.75, 230.24)	-4.24 (-6.10, -2.34)	-4.03 (-5.81, -2.22)
High-income North America	62.20 (52.99, 72.42)	18.58 (15.27, 22.16)	1559.16 (1358.54, 1765.61)	489.24 (412.74, 568.57)	-1.68 (-2.09, -1.28)	-1.74 (-2.08, -1.40)
North Africa and Middle East	81.28 (68.37, 95.16)	50.54 (40.61, 61.16)	2154.47 (1855.89, 2473.90)	1305.66 (1062.58, 1568.06)	-0.60 (-0.95, -0.25)	-0.52 (-0.86, -0.18)
Oceania	46.93 (36.41, 59.22)	39.27 (30.71, 49.78)	1470.65 (1142.01, 1880.09)	1261.35 (988.62, 1598.45)	-0.29 (-1.13, 0.55)	-0.57 (-1.29, 0.16)
South Asia	41.05 (33.15, 49.20)	36.59 (29.99, 44.19)	1150.67 (959.85, 1367.29)	948.63 (782.92, 1124.56)	-0.27 (-0.98, 0.45)	-0.22 (-0.70, 0.26)
Southeast Asia	39.61 (32.63, 45.98)	36.49 (30.20, 43.01)	1057.39 (885.58, 1218.13)	984.33 (824.69, 1167.94)	-3.41 (-4.00, -2.82)	-3.38 (-3.95, -2.82)

Southern Latin America	35.82 (30.84, 41.39)	11.66 (9.88, 13.65)	1015.96 (880.46, 1152.24)	338.60 (292.08, 387.23)	-1.76 (-4.82, 1.40)	-1.64 (-4.66, 1.47)
Southern Sub-Saharan Africa	24.70 (20.14, 29.68)	15.38 (12.76, 18.33)	687.79 (582.88, 807.23)	441.19 (369.23, 520.36)	-3.57 (-4.87, -2.25)	-3.64 (-4.71, -2.55)
Tropical Latin America	48.41 (41.61, 55.73)	16.02 (13.15, 19.21)	1324.19 (1159.37, 1490.65)	441.45 (368.85, 528.07)	-5.26 (-6.50, -3.99)	-5.03 (-6.17, -3.88)
Western Europe	57.02 (48.78, 66.15)	12.02 (10.02, 14.26)	1343.97 (1172.33, 1520.55)	298.39 (254.89, 344.38)	-0.24 (-1.96, 1.51)	-0.22 (-2.11, 1.71)
Western Sub-Saharan Africa	8.25 (6.13, 10.53)	7.89 (5.94, 9.88)	220.17 (166.28, 281.09)	212.47 (160.24, 266.02)	-5.90 (-7.47, -4.31)	-5.56 (-7.16, -3.94)

Supplementary Table 3. Global Distribution and EAPC of IHD burden attributable to smoking (male)

The ASDR and ASRDALYs per 100,000 population of IHD attributable to smoking among males across global regions, SDI levels, and specific geographic regions for 1990 and 2021 are summarized. Temporal trends from 1990 to 2021 are represented by the EAPC of ASDR and ASRDALYs, with corresponding 95% CI. Positive EAPC values indicate an increasing trend, while negative EAPC values denote a decreasing trend

Location	Both			Females			Males		
	Time Point	APC (95% CI)	p	Time Point	APC (95% CI)	p	Time Point	APC (95% CI)	p
Global	1990 -1994	0.21 (-0.17, 0.62)	0.216	1990 -1995*	-1.92 (-2.15, -1.52)	0.001	1990 -1994	0.38 (-0.02, 0.81)	0.057
	1994 -1998*	-2.74 (-3.30, -2.34)	<0.001	1995 -1998*	-3.60 (-3.97, -2.89)	<0.001	1994 -1998*	-2.72 (-3.30, -2.26)	<0.001
	1998 -2003*	-1.01 (-1.35, -0.39)	0.001	1998 -2003*	-2.44 (-3.32, -1.78)	<0.001	1998 -2003*	-0.77 (-1.11, -0.10)	0.030
	2003 -2021*	-2.07 (-2.12, -2.02)	<0.001	2003 -2009*	-3.64 (-4.25, -3.20)	<0.001	2003 -2021*	-1.93 (-1.99, -1.87)	<0.001
				2009 -2021*	-2.95 (-3.07, -2.78)	<0.001			
				2012 -2019*	-1.75 (-2.06, -0.40)	0.003			
				2019 -2021*	-0.90 (-1.63, -0.52)	<0.001			
Low SDI	1990 -1998*	0.30 (0.08, 0.55)	0.007	1990 -1995	-0.02 (-0.53, 1.09)	0.988	1990 -1998*	0.47 (0.07, 0.86)	0.040
	1998 -2006*	-1.65 (-1.98, -1.43)	<0.001	1995 -2002*	-1.14 (-1.67, -0.66)	0.016	1998 -2006	-1.50 (-1.81, 0.76)	0.164
	2006 -2014	-0.29 (-0.50, 0.10)	0.102	2002 -2010*	-1.98 (-2.65, -1.66)	0.008	2006 -2015	-0.17 (-1.81, 0.07)	0.106
	2014 -2021*	-1.29 (-1.62, -1.05)	<0.001	2010 -2013	2.78 (-1.28, 3.48)	0.054	2015 -2018	-1.34 (-1.74, 0.30)	0.157
				2013 -2018*	-4.12 (-4.89, -3.50)	0.046	2018 -2021	-0.26 (-0.98, 0.62)	0.535
				2018 -2021*	-2.29 (-3.34, -0.78)	<0.001			
Low-middle SDI	1990 -1997	-0.39 (-0.61, 0.10)	0.075	1990 -1998*	-1.29 (-1.88, -0.96)	0.019	1990 -2005	-0.36 (-0.63, 0.02)	0.054
	1997 -2005	-0.80 (-1.33, 0.37)	0.064	1998 -2005*	-2.31 (-3.08, -0.79)	0.004	2005 -2014	0.76 (-0.79, 1.19)	0.301
	2005 -2012	0.46 (-0.09, 0.75)	0.051	2005 -2010	-1.60 (-2.15, 0.48)	0.094	2014 -2017	-1.30 (-1.88, 1.10)	0.419
	2012 -2021*	-1.25 (-1.41, -1.10)	<0.001	2010 -2014	0.59 (-2.78, 1.35)	0.156	2017 -2021	-0.68 (-1.16, 0.19)	0.101
				2014 -2021*	-2.37 (-2.75, -1.85)	<0.001			
Middle SDI	1990 -1995*	-0.55 (-0.73, -0.32)	0.008	1990 -1996*	-1.54 (-1.65, -1.40)	<0.001	1990 -2001*	-0.78 (-0.96, -0.67)	0.010

	1995 -2000*	-1.37 (-1.78, -1.15)	<0.001	1996 -1999*	-2.98 (-3.17, -2.57)	<0.001	2001 -2004	0.32 (-0.50, 0.62)	0.322
	2000 -2004	-0.14 (-0.80, 0.24)	0.267	1999 -2004*	-2.03 (-2.17, -1.73)	<0.001	2004 -2007*	-1.17 (-1.54, -0.32)	0.031
	2004 -2007*	-1.49 (-1.73, -0.24)	0.014	2004 -2007*	-3.87 (-4.11, -3.44)	<0.001	2007 -2012	0.02 (-0.53, 0.62)	0.701
	2007 -2012	-0.30 (-1.25, 0.03)	0.061	2007 -2011*	-2.45 (-2.64, -2.11)	<0.001	2012 -2021*	-1.50 (-1.69, -1.35)	<0.001
	2012 -2019*	-1.75 (-2.06, -0.40)	0.003	2011 -2017*	-2.95 (-3.30, -2.83)	<0.001			
	2019 -2021*	-0.90 (-1.63, -0.52)	<0.001	2017 -2021*	-1.81 (-2.09, -1.39)	<0.001			
High-middle SDI	1990 -1994*	5.08 (4.12, 6.15)	<0.001	1990 -1994*	2.38 (1.64, 3.22)	<0.001	1990 -1994*	4.95 (3.99, 6.01)	<0.001
	1994 -1998*	-3.63 (-5.06, -2.61)	<0.001	1994 -1998*	-2.42 (-3.49, -1.58)	0.006	1994 -1998*	-3.75 (-5.19, -2.72)	<0.001
	1998 -2003*	1.73 (0.77, 3.06)	<0.001	1998 -2004*	0.94 (0.37, 1.68)	0.022	1998 -2003*	1.60 (0.66, 2.91)	<0.001
	2003 -2021*	-3.47 (-3.62, -3.34)	<0.001	2004 -2011	-3.08 (-3.43, -0.03)	0.050	2003 -2021*	-3.52 (-3.66, -3.38)	<0.001
				2011 -2017*	-4.44 (-5.52, -3.10)	<0.001			
				2017 -2021*	-3.11 (-4.05, -1.46)	<0.001			
High SDI	1990 -1994*	-3.80 (-4.21, -3.08)	<0.001	1990 -1994*	-3.79 (-4.35, -2.93)	<0.001	1990 -1994*	-3.92 (-4.23, -3.51)	<0.001
	1994 -2004*	-4.78 (-5.33, -4.65)	<0.001	1994 -2001*	-4.74 (-5.40, -4.50)	<0.001	1994 -2001*	-5.05 (-5.40, -4.90)	<0.001
	2004 -2012*	-4.22 (-4.45, -3.73)	<0.001	2001 -2010*	-5.43 (-5.87, -3.17)	<0.001	2001 -2010*	-4.44 (-4.60, -4.28)	<0.001
	2012 -2021*	-2.39 (-2.54, -2.18)	<0.001	2010 -2021*	-3.05 (-3.21, -2.86)	<0.001	2010 -2014*	-3.38 (-3.96, -2.81)	<0.001
							2014 -2021*	-2.26 (-2.42, -2.01)	<0.001

Supplementary Table 4. Trends in ASRDALYs of IHD attributable to smoking by sex and SDI regions from 1990 to 2021

The joinpoint regression results for ASRDALYs (per 100,000 population) trends of IHD attributable to smoking across SDI regions and stratified by sex from 1990 to 2021 were shown. The time points represent the breakpoints where significant changes in the trends occurred, with APC (annual percent change) and 95% CI provided. Positive APC values indicate an increasing trend, while negative APC values denote a decreasing trend. Statistically significant APCs ($p<0.05$) are marked with *, which means significantly significant increasing or decreasing trend, while $p>0.05$ suggests no significant change (stable trend).

© 2025 Wang Y. et al.
